# Morphological characterization and genetic diversity analysis of Tunisian durum wheat (*Triticum turgidum* var. *durum*) accessions

**DOI:** 10.1186/s12863-021-00958-3

**Published:** 2021-02-03

**Authors:** Maroua Ouaja, Bochra A. Bahri, Lamia Aouini, Sahbi Ferjaoui, Maher Medini, Thierry C. Marcel, Sonia Hamza

**Affiliations:** 1 Institut National Agronomique de Tunis, Université de Carthage, 43 Avenue Charles-Nicolle, Tunis, 1082 Tunisie; 2 Department of Plant Pathology and Institute of Plant Breeding, Genetics, and Genomics, University of Georgia, Griffin, GA 30223 USA; 3 Centre Régional de Recherches en Grandes Cultures (CRRGC), Route de Tunis, BP, 350 Beja, Tunisie; 4 Banque Nationale des gènes, Boulevard du Leader Yasser Arafat Z. I Charguia 1, Tunis, 1080 Tunisie; 5 UMR BIOGER, INRA, AgroParisTech, Université Paris-Saclay, Thiverval-Grignon, France

**Keywords:** Durum wheat, Local landraces, Landrace characterization, Phenotypic diversity, Genetic diversity, Population structure

## Abstract

**Background:**

Tunisia is considered a secondary center of diversification of durum wheat and has a large number of abandoned old local landraces. An accurate investigation and characterization of the morphological and genetic features of these landraces would allow their rehabilitation and utilization in wheat breeding programs. Here, we investigated a diverse collection of 304 local accessions of durum wheat collected from five regions and three climate stages of central and southern Tunisia.

**Results:**

Durum wheat accessions were morphologically characterized using 12 spike- and grain-related traits. A mean Shannon-Weaver index (*H′*) of 0.80 was obtained, indicating high level of polymorphism among accessions. Based on these traits, 11 local landraces including Mahmoudi, Azizi, Jneh Khotifa, Mekki, Biskri, Taganrog, Biada, Badri, Richi, Roussia and Souri were identified. Spike length (*H′* = 0.98), spike shape (*H′* = 0.86), grain size (*H′* = 0.94), grain shape (*H′* = 0.87) and grain color (*H′* = 0.86) were the most polymorphic morphological traits. The genetic diversity of these accessions was assessed using 10 simple sequence repeat (SSR) markers, with a polymorphic information content (PIC) of 0.69. Levels of genetic diversity were generally high (*I* = 0.62; *He* = 0.35). In addition, population structure analysis revealed 11 genetic groups, which were significantly correlated with the morphological characterization. Analysis of molecular variance (AMOVA) showed high genetic variation within regions (81%) and within genetic groups (41%), reflecting a considerable amount of admixture between landraces. The moderate (19%) and high (59%) levels of genetic variation detected among regions and among genetic groups, respectively, highlighted the selection practices of farmers. Furthermore, Mahmoudi accessions showed significant variation in spike density between central Tunisia (compact spikes) and southern Tunisia (loose spikes with open glume), may indicate an adaptation to high temperature in the south.

**Conclusion:**

Overall, this study demonstrates the genetic richness of local durum wheat germplasm for better in situ and ex situ conservation and for the subsequent use of these accessions in wheat breeding programs.

**Supplementary Information:**

The online version contains supplementary material available at 10.1186/s12863-021-00958-3.

## Background

Durum wheat (*Triticum turgidum* var. *durum* Desf.) is a tetraploid species (2n = 4x = 28, AABB) that originated and domesticated in the Fertile Crescent and spread within the Mediterranean region through different dispersal [1, 2], reaching the Iberian Peninsula through Northern Africa around 7000 BC [3]. Since then, durum wheat has gained commercial importance. Today, durum wheat is cultivated worldwide, especially in the Mediterranean Basin, which is considered as the center of diversification and production of durum wheat [4, 5]. The Mediterranean Basin is characterized by highly variable environments, ranging from warm and dry to cool and wet climates [6]. Durum wheat accessions collected from the Mediterranean region exhibit higher genetic diversity than those collected from other regions of the world [7].

Within the Mediterranean region, Tunisia is one of the main centers of diversity of durum wheat [8, 9]. Old Tunisian durum wheat cultivars are known by their high level of genetic diversity and their specific adaptability to North African drylands [10]. Despite their notable genetic diversity, Tunisian landraces have been progressively abandoned since the first decade of the twentieth century and replaced by improved, high-yielding and genetically uniform semi-dwarf cultivars (known as “modern varieties”) developed through international breeding programs [11, 12]. This has led to a significant reduction in the genetic diversity of local durum wheat [13, 14]. Nonetheless, the genetic diversity of durum wheat could be preserved by: (1) characterizing the remaining durum wheat landraces; (2) re-introducing these landraces into breeding programs; and (3) protecting these landraces through effective conservation strategies. Therefore, the genetic and morpho-phenological characterization of landraces, which are either sparsely cultivated under the current farming system or stored in gene banks, would allow the identification of unexplored sources of genetic diversity that may be important for adaptation to several biotic and abiotic stresses [7, 15, 16]. The availability of landraces for breeding programs may also have particular relevance for breeding cultivars suitable for suboptimal and marginal environments such as the Mediterranean Basin, where durum wheat and other crop species are largely cultivated under unstable and limited water conditions, which cause considerable yield fluctuations [17, 18].

Previously, the agro-morphological evaluation of Tunisian durum wheat accessions using quantitative and qualitative spike-related traits, mostly concerning the grains, revealed high morphological diversity within the Tunisian durum wheat landraces [19, 20], and more than 35 durum wheat landraces were recorded [13]. However, few studies have been conducted to analyze the morphological and genetic features of durum wheat simultaneously. Moreover, the correlation between genetic population structure and morphological aspects of durum wheat has not been investigated to date. Previously, analysis of the level of genetic diversity in Tunisian durum wheat germplasm using amplified fragment length polymorphism (AFLP) and simple sequence repeat (SSR) markers revealed an important polymorphism within cultivars [10]. More recently, investigation of the genetic diversity and population structure of 196 durum wheat landrace accessions (including Tunisian and North African accessions) using diversity array technology sequencing (DArTseq)-based markers showed that genetic variation was higher among landraces than within landraces, and the Tunisian and North African landraces showed remarkable genetic similarity [21]. Furthermore, Slim et al. [22] evaluated the genetic structure of Tunisian durum wheat germplasm, and suggested the existence of five subpopulations with a strong genetic stratification from the north to the south of Tunisia, probably due to the prevalence of modern cultivars in the north. By tracing the history of cultivation, Tunisian durum wheat germplasm collections have been divided into three distinct categories: traditional varieties or old landraces, old cultivars (cultivated up to 1970s) and modern cultivars (cultivated up to present) [10, 13, 22]. Since traditional local landraces have been derived either from artificial selection of traditional farming systems or from natural adaptation to adverse growing conditions, these landraces might harbor key traits for breeding programs.

Taking into account the value of traditional Tunisian durum wheat landraces, we aimed to: (i) evaluate the genetic diversity and population structure of 304 Tunisian durum wheat accessions collected from central and southern Tunisia using SSR markers; (ii) study the phenotypic diversity of these accessions, based on the morphological characterization of spike- and grain-related traits; and (iii) analyze the relationship between genetic and phenotypic variation.

## Results

### Morphological characterization of Tunisian durum wheat accessions

#### Phenotypic diversity and morphological characterization

The Shannon-Weaver index (*H′*) revealed a high morphological diversity among durum wheat accessions (*H′* = 0.80) (Table 1). The most polymorphic characters were spike length (SL; *H′* = 0.98), grain size (GSz; *H′* = 0.94), grain shape (GSp; *H′* = 0.87), grain color (GC; *H′* = 0.86) and spike shape (SS; *H′* = 0.86), while the least polymorphic trait was spike color (SC; *H′* = 0.53).

**Table 1:** Shannon-Weaver index (*H′*) estimated on the 304 Tunisian durum wheat accessions for five regions and for three climate stages

		Phenotypic traits												
		SS	SL	AL	SC	NS	GlC	GN	GSp	GSz	GC	AC	SD	Mean ***H′***
**Collection**		0.86	0.98	0.79	0.53	0.83	0.84	0.69	0.87	0.94	0.86	0.64	0.74	**0.80**
**Regions**	Sousse	0	0	0	0	0	0	0	0	0	0	0	0	0.00
	Mahdia	0.52	0.99	0.56	0.00	0.63	0.48	0.62	0.63	0.48	0.74	0.73	0.65	0.58
	Kairouan	0.85	0.98	0.57	0.48	0.96	0.97	0.62	0.98	0.88	0.75	0.12	0.70	0.74
	Gabes	0.44	0.49	0.50	0.25	0.63	0.50	0.57	0.56	0.43	0.73	0.61	0.65	0.53
	Medenine	0.60	0.71	0.86	0.53	0.59	0.53	0.78	0.86	0.63	0.62	0.71	0.47	0.66
	**Mean**	0.54	0.69	0.55	0.30	0.61	0.55	0.55	0.65	0.56	0.62	0.47	0.53	**0.55**
**Climate stages**	LSA	0.48	0.00	0.92	0.63	0.61	0.41	0.62	0.61	0.62	0.74	0.67	0.79	0.59
	MA	0.68	0.38	0.80	0.87	0.62	0.56	0.70	0.88	0.54	0.89	0.71	0.65	0.69
	HA	0.85	0.48	0.97	0.58	0.96	0.96	0.63	0.98	0.88	0.75	0.12	0.68	0.74
	**Mean**	0.67	0.29	0.90	0.69	0.73	0.64	0.65	0.82	0.68	0.79	0.50	0.71	**0.67**

*SS* spike shape, *SL* spike length, *AL* awn length, *SC* spike color, *NS* number of spikelets/spike, *GlC* glume color, *GN* number of grains/spikelet, *GSp* grain shape, *GSz* grain size, *GC* grain color, *AC* awn color, *SD* spike density, *LSA* Low Semi-Arid (Sousse and Mahdia), *MA* Mid-Arid (Gabes and Medenine), *HA* Higher-Arid (Kairouan)

The 304 durum wheat accessions investigated in this study were grouped into 11 landraces, namely Azizi, Jneh Khotifa, Taganrog, Mekki, Richi, Souri, Roussia, Badri, Biskri, Biada and Mahmoudi, recorded in the catalog of durum wheat landraces cultivated in Tunisia [13]. These landraces were characterized by 12 specific morphological traits, based on the International Plant Genetic Resources Institute (IPGRI) [23] and International Union for the Protection of New Varieties of Plants (UPOV) [24] (Table S1, Table S2). All 12 spike and grain characteristics were almost homogeneous among accessions of the same landrace. This was supported by the Shannon-Weaver index (*H′*), which was relatively low for each landrace, ranging from 0.00 (Badri and Jneh Khotifa) to 0.23 (Richi), with an overall mean of 0.11 (Table S3). For instance, Mahmoudi accessions had particularly large spikes with sub-pyramidal shape, very long awns and large grains, whereas spikes of Azizi accessions were rectangular and very flat. Biskri accessions had fusiform and large spikes. The spike color, length and shape varied among the studied accessions from dark to light and from short to long spikes. For example, Badri spikes were very short and thick with a greyish color, whereas Biada spikes and awns were very light (white) in color. Souri and Roussia were both characterized by tight, red-colored spikes with a distinct spike shape, i.e., either rectangular (Souri) or cylindrical (Roussia). Souri and Roussia landraces were also characterized by a distinct orange colored grain. Interestingly, Richi accessions showed a unique feathery spike, while Mekki accessions were characterized by short and dense spikes with parallel edges. Finally, Taganrog accessions were characterized by white colored spikes with black stains, while Jneh Khotifa accessions showed very dark (black to purple), long and dense spikes and awns.

#### Principal component analysis (PCA)

**Fig. 1:**
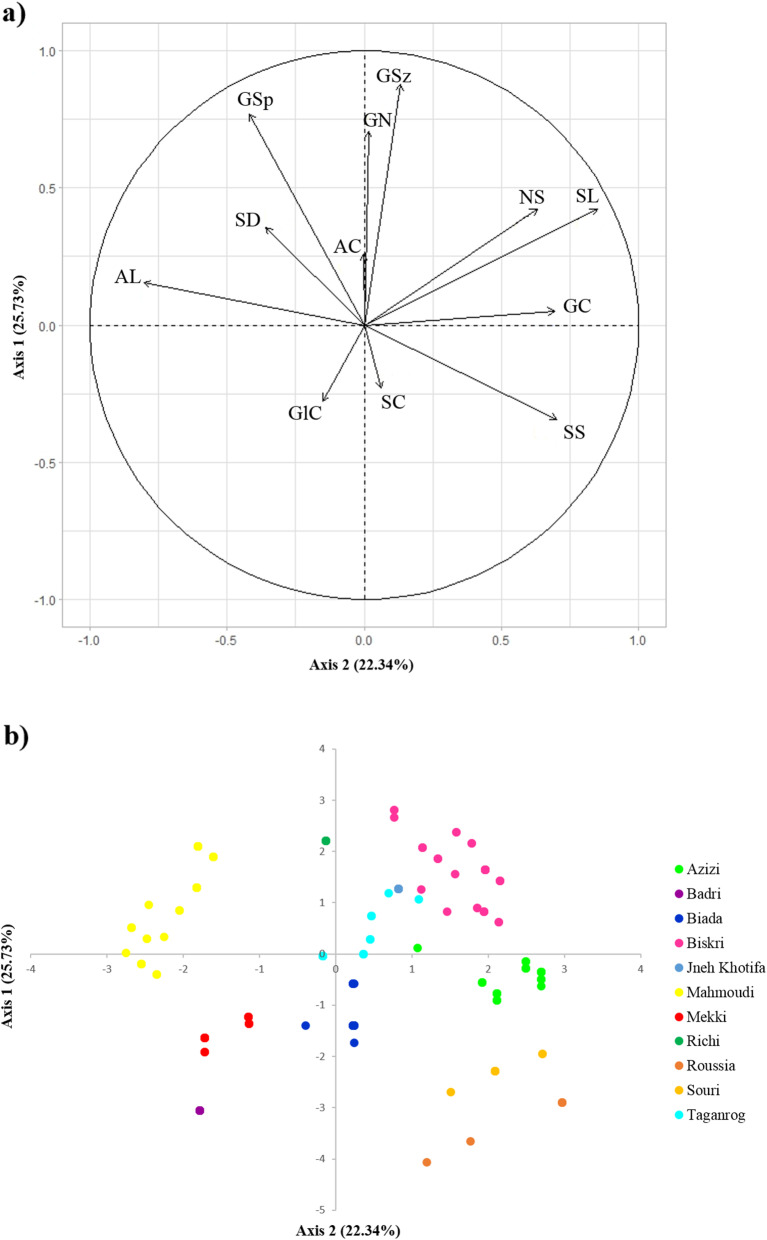
Principal component analysis plot depicting 11 durum wheat landraces within 304 Tunisian accessions using 12 morphological traits under GenAlEx (version 6.501) [25]; (**a**) Projection of the 12 variables on the PCA plot axes. SS: spike shape, SL: spike length, AL: awn length, SC: spike color, NS: number of spikelets/spike, GlC: glume color, GN: number of grains/spikelet, GSp: grain shape, GSz: grain size, GC: grain color, AC: awn color, SD: spike density; (**b**) Projection of the 304 accessions on the PCA plot axes. Accessions were color-coded according to their landraces nomenclature, as identified with the morphological characterization

PCA of 12 spike and grain morphological traits of 304 durum wheat accessions showed that PC1 and PC2 axes accounted for 25.73 and 22.34% of the total genetic variation in these traits, respectively (Fig. 1). PC1 was mostly associated with SS, SL, number of spikelet per spike (NS), grain color (GC) and awn length (AL), whereas PC2 was mainly associated with GSp, GSz and grain number per spikelet (GN) (Fig. 1a). The color-coding of accessions in the two-dimensional PCA plot (PC1 vs. PC2) showed a good correspondence between the morphological trait-based grouping and landrace denomination (Fig. 1b), and accessions belonging to the same landrace were included in the same PCA subgroup. Biskri, Jneh Khotifa and Taganrog accessions grouped together, showed positive correlation with both PC1 and PC2 and shared similar spike characteristics, such as SL (mostly long spikes), high GN (> 3), black awn color (AC) and AL longer than the spike. Azizi accessions were grouped into a distinct subgroup, mainly characterized by rectangular medium-sized spikes with a tan color. Mahmoudi accessions also formed a distinct subgroup, mainly characterized by unique pyramid-shaped spikes. Accessions of Souri and Roussia formed almost a single subgroup characterized by red-colored loose and long spikes as well as red colored glumes and awns. Landraces Badri and Mekki formed distinct subgroups negatively correlated to PC1 and PC2, and both subgroups were mainly characterized by short spikes with a low to intermediate GN. Biada and Richi accessions were grouped mainly in the center of the plot and were particularly characterized by white-colored spikes, glumes and awns (Table S2). Overall, PC1 and PC2 could separate all landraces, based on 12 spike- and grain-related morphological traits; the only exceptions were the groups of Roussia and Souri landraces and Biskri, Jneh Khotifa and Taganrog landraces, which could not be distinguished based on SL and SC. Thus, additional morphological traits, such as glume form, were considered to classify the latter landraces into distinct subgroups (Table S2).

### Genetic diversity and population structure of Tunisian durum wheat accessions

#### SSR polymorphism

Ten SSR markers were used in this study to analyze the genetic diversity and population structure of Tunisian durum wheat accessions. These SSR markers were mapped onto eight different chromosomes and therefore were considered largely independent (Table 2, Table S4). The percentage of missing data was low (< 10%) for each locus. All 10 SSR markers amplified a total of 99 alleles and from 302 accessions, 188 multilocus genotypes (MLGs) were identified. The accumulation curve (Figure S1), showed that these SSR markers were able to reach the maximal range of differentiation among the MLGs. The number of different alleles per locus (Na) varied from 4 (Xgpw2103) to 16 (Xgwm413), with an average Na of 9.9 across all loci. Overall, the PIC value was 0.690. The highest PIC value was obtained for Xgwm413 (0.851), whereas the lowest PIC value was obtained for Xgpw2103 (0.448). The Shannon’s information index (*I*) value was the highest for Xgwm413 (2.182) and the lowest for Xgpw2103 (0.781). The fixation index (*Fis*) was approximately equal to 1 for each locus, except Xgwm495 (*Fis* = − 0.373), for which a high PIC value was observed (0.659). Pairwise genetic differentiation (F*st*) ranged from 0.201 (Xgwm495) to 0.688 (Xgpw7148).

**Table 2:** Polymorphism level of 10 Simple Sequence Repeats (SSR) markers used on 302 Tunisian durum wheat accessions

Locus	N	Na	***I***	***Fis***	F***st***	PIC
**Xgwm413**	302	16	2.182	0.987	0.337	0.851
**Xgpw7148**	302	8	1.294	1.000	0.688	0.665
**Xgwm495**	300	11	1.614	−0.373	0.201	0.659
**Xgwm193**	298	10	1.338	1.000	0.577	0.621
**Xgpw2239**	302	8	1.695	1.000	0.424	0.773
**Xgwm285**	299	12	1.832	0.965	0.624	0.805
**Xgpw4082**	282	7	1.324	1.000	0.737	0.632
**Xgpw4004**	278	11	1.546	1.000	0.589	0.740
**Xgpw2103**	291	4	0.781	1.000	0.523	0.448
**Xgwm372**	275	12	1.643	0.988	0.491	0.705
**Total**	292.9 (3.378)	9.9 (1.048)	1.525 (0.118)	0.857 (0.137)	0.519 (0.052)	0.690

*N* Samples size, *Na* Number of Alleles, *I* Shannon’s Information Index, *Fis* Inbreeding coefficient within individuals, *Fst* Inbreeding coefficient within genetic groups, *PIC* Polymorphic Information Content

#### Analysis of population structure and relationship with morphological characterization

**Fig. 2:**
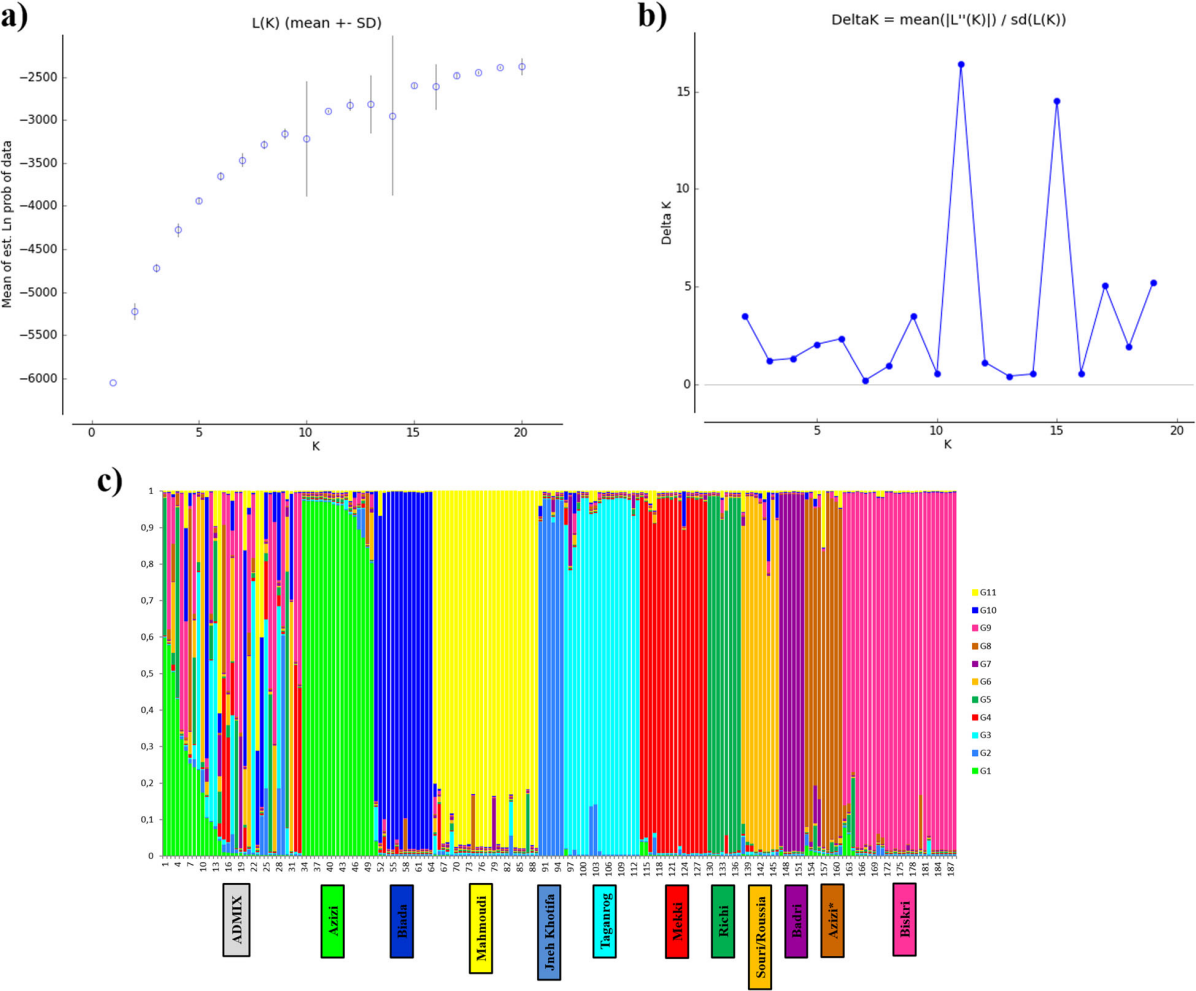
Population structure analysis of 302 Tunisian durum wheat accessions genotyped with 10 SSR markers: (**a**) Plot of mean posterior probability (ln P(D)) values per cluster (K); (**b**) delta-K analysis of Ln P(D), based on 10 replicates per K, for K ranging from 1 to 20, with a burn-in period of 100,000 and Monte Carlo Markov Chain replicates of 100,000 iterations; (**c**) Membership coefficient bar plot displaying population structure at K = 11 for 302 Tunisian durum wheat accessions genotyped with 10 SSR markers inferred from STRUCTURE [26]. Each MLG is represented by a vertical line and they are ordered by color-coded genetic group (G1 to G11). For each genetic group, corresponding durum wheat landrace is mentioned. * Azizi landrace was divided into two genetic group G1 and G8

We analyzed the population structure on 188 MLGs. The maximum likelihood (LnP(K)) and delta K (ΔK) methods indicated that the most likely number of genetic groups (K) was 11 (Fig. 2a, b). The estimated genetic group membership coefficient of each accession at K = 11 is shown in the population structure plot (Fig. 2c).

Overall, each genetic group corresponded to a single landrace. The genetic groups G2, G3, G4, G5, G7, G9, G10 and G11 corresponded to Jneh Khotifa, Taganrog, Mekki, Richi, Badri, Beskri, Biada and Mahmoudi landraces, respectively. Moreover, a significant correlation was detected between the genetic distance matrix and morphological distance matrix (*P* = 0.01; R_xy_ = 0.435). However, a discrepancy between the genetic grouping and the morphological characterization was observed for Azizi, Souri and Roussia landraces; Azizi landraces were grouped by STRUCTURE into two different genetic groups G1 and G8, while Souri and Roussia landraces were grouped together into one genetic group (G6), despite their distinct morphological characteristics.

A total of 41 admixed individuals were observed in the collection. The admixture was mainly obtained between G6 (Roussia and Souri) and G10 (Biada) (representing 14.6% of the admixed genotypes), followed by G1 (Azizi) and G9 (Beskri) (representing 9.7%). Mahmoudi (G11), Beskri (G9) and admixed genotypes were the most frequent (representing 23.8, 12.2 and 14% of the entire collection, respectively), followed by Azizi (G1), Taganrog (G3), Mekki (G4), Badri (G7) and Biada (G10) (each accounting for approximately 8% of the entire collection). However, Jneh Khotifa (G2), Richi (G5), Roussia and Souri (G6) and Azizi (G8) were the least frequent, each accounting for 3% of the entire collection.

#### Analysis of diversity indices and molecular variance

The 11 groups identified by STRUCTURE analysis presented different levels of genetic diversity (Table 3). Group G6 showed the highest level of genetic diversity, while G7 represented the lowest level. The number of effective alleles per locus (*Ne*) ranged from 1.152 (G7) to 2.379 (G6). Genetic groups with the highest number of MLGs were G6 (100% of different MLGs), G8 (90%) and G3 (85.7%), while G7 and G11 had the lowest number of MLGs (27.2 and 34.7%, respectively). The percentage of polymorphism (*P*) ranged from 40% (G7) to 100% (G6 and G8). Shannon’s information index (*I*) varied from 0.166 (G7) to 0.937 (G6), with an average value of 0.620 across all accessions. In addition, G6 and G8 showed the highest number of private alleles (G6, *PA* = 7; G8, *PA* = 4), while G2 and G7 contained no private alleles (*PA* = 0) (Table S5). Groups G10 and G4 contained two diagnostic alleles (DA) each, while G3, G5 and G7 contained one DA each, with frequency > 70%. The fixation index (*Fis* ranged from 0.698 (G4) to 1.0 (G7), where observed heterozygosity (*Ho*) was 0.100 and null, respectively. Furthermore, analysis of variance (ANOVA) showed that 59% of the total genetic diversity was observed between distinct genetic groups, while 41% of the genetic diversity was explained by differences within each group (Table 4).

**Table 3:** Diversity indexes of 302 Tunisian durum wheat accessions sorted by genetic groups as defined by STRUCTURE [26], by regions and by climate stages

		Acc	MLG	S	Ne	***I***	***Ho***	***He***	***Fis***	P (%)	PA	***Nm***	L	DA^b^	^a^
**Genetic groups**	**ADMIX**	41	33	13	3.904 (0.387)	1.522 (0.107)	0.088 (0.080)	0.721 (0.027)	0.871 (0.118)	100	6		–	–	–
	**G1**	24	17	5	1.830 (0.331)	0.627 (0.156)	0.033 (0.029)	0.334 (0.078)	0.869 (0.118)	90	3		100% Azizi	–	–
	**G10**	21	14	4	1.591 (0.229)	0.431 (0.144)	0.105 (0.100)	0.261 (0.088)	0.726 (0.201)	60	1		100% Biada	179 (**Xgwm193**) 214 (**Xgpw4082**)	1.00 0.94
	**G11**	72	25	11	1.443 (0.205)	0.394 (0.144)	0.099 (0.099)	0.210 (0.079)	0.784 (0.180)	70	1		100% Mahmoudi	–	–
	**G2**	9	6	3	1.694 (0.186)	0.510 (0.127)	0.111 (0.099)	0.332 (0.080)	0.688 (0.236)	70	0		100% JK	–	–
	**G3**	21	18	1	1.948 (0.341)	0.629 (0.166)	0.100 (0.100)	0.369 (0.087)	0.767 (0.195)	70	1		100% Taganrog	216 (**Xgpw4082**)	0.71
	**G4**	26	16	–	1.567 (0.215)	0.455 (0.138)	0.100 (0.100)	0.266 (0.082)	0.698 (0.234)	60	1		100% Mekki	193 (**Xgwm413**) 321 (**Xgwm372**)	0.84 0.84
	**G5**	10	8	2	1.487 (0.196)	0.424 (0.126)	0.100 (0.100)	0.244 (0.073)	0.768 (0.194)	70	2		100% Richi	224 (**Xgpw4004**)	0.9
	**G6**	9	9	3	2.379 (0.274)	0.937 (0.110)	0.078 (0.078)	0.529 (0.051)	0.893 (0.107)	100	7		41% Roussia 59% Souri	–	–
	**G7**	22	6	3	1.152 (0.077)	0.166 (0.075)	0.000 (0.000)	0.103 (0.049)	1.000 (0.000)	40	0		100% Badri	232 (**Xgpw4082**)	1.0
	**G8**	10	9	3	1.905 (0.183)	0.733 (0.103)	0.010 (0.010)	0.428 (0.056)	0.962 (0.038)	100	4		100% Azizi	–	–
	**G9**	37	27	3	1.799 (0.219)	0.609 (0.139)	0.043 (0.043)	0.362 (0.080)	0.911 (0.080)	80	2		100% Biskri	–	–
	**Total**	302	188	–	1.892 (0.092)	0.620 (0.047)	0.072 (0.022)	0.346 (0.024)	0.835 (0.042)	75,83 (5,43)	–	0.259 (0.079)	–	–	–
**Regions**	**Gabes**	38	31	3	3.031 (0.491)	1.296 (0.122)	0.056 (0.047)	0.610 (0.045)	0.879 (0.11)	100	17			171 (**Xgwm193**)	0.71
	**Kairouan**	67	25	6	2.707 (0.405)	1.048 (0.136)	0.042 (0.041)	0.563 (0.054)	0.880 (0.117)	100	2			–	–
	**Mahdia**	27	21	4	2.883 (0.293)	1.275 (0.102)	0.081 (0.077)	0.619 (0.04)	0.873 (0.121)	100	11			–	–
	**Mednine**	22	7	3	1.960 (0.158)	0.790 (0.099)	0.095 (0.095)	0.45 (0.056)	0.851 (0.149)	100	1			–	–
	**Sousse**	9	7	1	1.366 (0.185)	0.305 (0.125)	0.100 (0.100)	0.183 (0.073)	0.691 (0.218)	50	1			191 (**Xgwm413**) 223 (**Xgwm285**) 224 (**Xgpw4004**)	0.88 0.77 1.00
	**Total**	163	91	17	2.389 (0.168)	0.943 (0.073)	0.075 (0.033)	0.485 (0.033)	0.851 (0.059)	90 (10)	–	1.037 (0.239)		–	–
**Climate stages**	**High-arid**	67	25	6	2.707 (0.405)	1.050 (0.136)	0.042 (0.041)	0.563 (0.054)	0.880 (0.117)	100	2			–	–
	**Low semi-arid**	36	28	5	3.006 (0.356)	1.283 (0.113)	0.086 (0.083)	0.622 (0.046)	0.870 (0.126)	100	12			–	–
	**Mid-arid**	60	38	6	3.174 (0.433)	1.318 (0.109)	0.070 (0.065)	0.642 (0.039)	0.870 (0.122)	100	19			–	–
	**Total**	163	91	17	2.962 (0.225)	1.216 (0.071)	0.066 (0.036)	0.609 (0.027)	0.874 (0.068)	100	–	3.813 (0.571)		–	–

*Acc* Number of accessions, *MLG* Number of Multi Locus Genotypes, *S* Number of sites, *Ne* Number of Effective Alleles, *I* Shannon’s Information Index, *Ho* Observed Heterozygosity, *He* Expected Heterozygosity, *Fis* Fixation Index, *P* Percentage of Polymorphic Loci, *PA* Number of Private Alleles, *Nm* gene flow, *L* Name of the landrace, *DA* Diagnostic alleles; ^a^: Frequence (0.7–1). ^b^: a DA is a rare allele with a frequence > 70% for a population or region and < 30% for the others

**Table 4:** Analysis of molecular variance (AMOVA) of Tunisian durum wheat accessions using 10 SSR markers by genetic groups as defined by STRCUTURE [26], by regions and by climate stages

	Source	df	SS	MS	Est. Var.	%
**Genetic groups**^a^	Among	10	1951.085	195.108	8.430	59
	Within	250	1471.172	5.885	5.885	41
	**Total**	260	3422.257	–	14.314	100
**Regions**	Among	4	353.123	88.281	2.605	19
	Within	158	1736.681	10.992	10.992	81
	**Total**	162	2089.804	–	13.597	100
**Climate stages**	Among	2	158.647	79.323	1.276	10
	Within	160	1931.157	12.070	12.070	90
	**Total**	162	2089.804	–	13.346	100

*df* degree of freedom, *SS* Sum of Squares, *MS* Mean Squares; %: pourcentage of variance

^a^Admixed genotypes were excluded from the analysis

#### Minimum spanning network (MSN) analysis

**Fig. 3:**
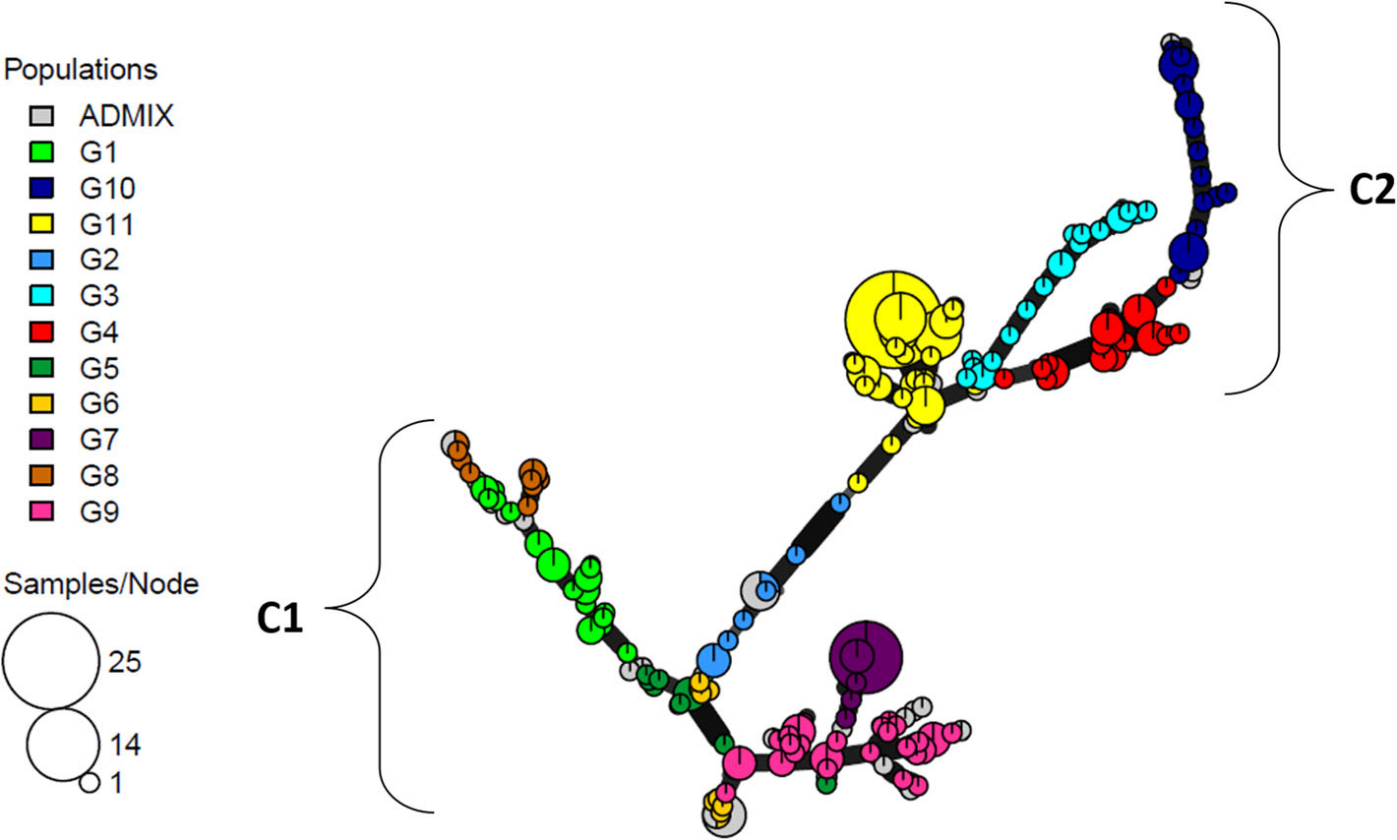
Minimum spanning network using Bruvo’s distance of 302 durum wheat accessions genotyped with 10 SSR markers, performed under R software. Each node represents a multilocus genotype (MLG) and the size of the node is proportional to the number of accessions representing the MLG. MLGs were color-coded according to their membership to a genetic group (G1 to G11) as defined by STRUCTURE [26] at K = 11. Admixed individuals were color-coded in grey. Edge widths represent relatedness

The genetic relatedness between genotypes was tested using MSN analysis, based on Bruvo’s distance. MSN separated all accessions into two main clusters (Fig. 3). Cluster C1 contained accessions belonging to Azizi (G1 and G8), Jneh Khotifa (G2), Richi (G5), Souri and Roussia (G6), Badri (G7) and Biskri (G9) landraces, while cluster C2 contained accessions belonging to Taganrog (G3), Mekki (G4), Biada (G10) and Mahmoudi (G11) landraces. In addition, the pairwise Nei’s genetic distances calculated between the 11 genetic groups were consistent with the results of MSN analysis (Table S6). The highest Nei’s genetic distance was recorded between G10 and G5 (2.416), followed by that between G10 and G7 (2.319). The lowest genetic distances were 0.421 registered between G1 and G8; and 0.630 registered between G3 and G11 and between G3 and G4 indicating that G1 and G8 as well as G3, G11 and G4 were genetically the most closely related groups. In addition, a morphological comparison between the network groupings revealed a significant difference (*P* < 0.05) between C1 and C2 in terms of SS, SL, AL, GC, GSp, NS, AC and glume color (GlC) (Table 5). Cluster C1 showed higher gene diversity (*He* = 0.740) and phenotypic diversity (*H′* = 0.77) than cluster C2 (*He* = 0.425, *H′* = 0.61) (Table S7 and S8). The values of SS and SL were higher in cluster C1 than in cluster C2, whereas C2 showed significantly higher AL and GSz than cluster C1 (Table 5).

**Table 5:** Means of morphological traits calculated for Azizi and Mahmoudi accessions from the center and the south of Tunisia and for all accessions from C1 and C2 clusters. Means with distinct letters show significant differences at 5% threshold between center and southern accessions

	Center		South		C1	C2
	AZ	MH	AZ	MH		
**SC**	1 ^a^	1 ^a^	1 ^a^	1 ^a^	1 ^a^	1 ^a^
**SS**	7 ^a^	1 ^b^	7 ^a^	1 ^b^	5–7 ^a^	3 ^b^
**SD**	3–5 ^a^	7 ^b^	3–5 ^a^	5 ^c^	7 ^a^	7 ^a^
**SL**	5 ^a^	1–3 ^b^	5 ^a^	1–3 ^b^	3–5 ^a^	1–3 ^b^
**AL**	3 ^a^	5 ^b^	3 ^a^	5 ^b^	3 ^a^	5 ^b^
**AC**	4 ^a^	3 ^a^	4 ^a^	3 ^a^	4 ^a^	3 ^b^
**NS**	3 ^a^	2 ^a^	2 ^a^	2 ^a^	2 ^a^	2 ^b^
**GlC**	1 ^a^	1 ^a^	1 ^a^	1 ^a^	1 ^a^	2 ^b^
**GC**	5 ^a^	1 ^b^	5 ^a^	1 ^b^	3 ^a^	1 ^b^
**GSp**	2 ^a^	3 ^b^	2 ^a^	3 ^b^	2 ^a^	3 ^b^
**GSz**	5 ^a^	7 ^b^	5 ^a^	7 ^b^	5 ^a^	5–7 ^a^
**GN**	2 ^a^	3 ^b^	2 ^a^	3 ^b^	2 ^a^	2 ^a^

Center: Mahdia, Sousse and Kairouan; South: Gabes and Medenine

*AZ* Azizi landrace (G1 and G8), *MH* Mahmoudi landrace (G11), *C1* Cluster 1 = G1, G2, G5, G6, G7, G8 and G9, *C2* Cluster 2 = G3, G4, G10 and G11, *SC* spike color, *SS* spike shape, *SD* spike density, *SL* spike length, *AL* awn length, *AC* awn color, *NS* number of spikelets/spike, *GlC* glume color, *GC* grain color, *GSp* grain shape, *GSz* grain size, *GN* number of grains/spikelet, *Hd* heading (days)

### Diversity analysis of Tunisian durum wheat accessions based on regions and climate stages

#### Analysis of morphological diversity among different regions and climate stages

The Shannon-Weaver index (*H′*) of 12 spike and grain related traits was compared among five regions (Sousse, Mahdia, Kairouan, Gabes and Medenine) and three different climate stages (low semi-arid, high-arid and mid-arid) (Table 1). Among all five regions, Kairouan showed the highest diversity index (*H′* = 0.74), followed by Medenine (*H′* = 0.66). Sousse showed a null diversity index, indicating no phenotypic variability between accessions in this region; notably, Richi was the only landrace identified in this region. The most polymorphic characteristics by regions were SL (*H′* = 0.69), GSp (*H′* = 0.65), GC (*H′* = 0.62) and NS (*H′* = 0.61). Among all three climate stages, the high-arid climate (represented by Kairouan) showed the highest diversity index (*H′* = 0.74), whereas the low semi-arid climate (represented by Mahdia and Sousse) showed the lowest diversity index (*H′* = 0.59). The most polymorphic characters by climate stages were AL (*H′* = 0.90), GSp (*H′* = 0.82), GC (*H′* = 0.79), and NS (*H′* = 0.73).

The polymorphism level of some morphological characteristics differed distinctly among regions, excluding Sousse where an overall homogeneity of morphological traits was recorded. The value of AC varied among regions from 0.12 (Kairouan) to 0.73 (Mahdia). Similarly, SL was the highest in Mahdia (0.99) and lowest in Gabes (0.49). Values of spike color (SC) and glume color (GC) indices were the highest in Medenine (0.53) and Kairouan (0.97), respectively, and lowest in Mahdia (0.00 and 0.48, respectively). Morphological traits were also variable from one climate stage to another. Values of SL and glume color were the highest in high-arid climate (0.48 and 0.96, respectively) and lowest in low semi-arid climate (0.0 and 0.41, respectively). By contrast, AC was the lowest in high-arid climate (0.12) and the highest in mid-arid climate (0.71). However, no variation was observed among regions for GC and among climates for GN.

In addition, a dominant phenotypic class of some morphological traits was observed among regions (within more than 70% of accessions), except Sousse, which did not show any variation in morphological traits (Table S9). In Gabes, the majority of accessions showed long spikes (SL > 9 cm; 84%), with light color (92%) and cylindrical shape (79%), awns shorter than the spike (84%), moderately elongated grain shape (82%), small grains (GSz < 0.3 cm) (82%) and an intermediate number of grains per spikelet (GN = 2–3; 79%), whereas accessions with medium length spikes (SL: 6–9 cm) were dominant in Medenine (73%). In Mahdia, the majority of accessions showed spikes with AL equal to the spike (72%) and small GSz (78%). However, most of the accessions in Kairouan had spikes with AL longer than the spike (72%). Among different climate stages, the mid-arid was dominated by accessions with small grains (GSz < 0.3 cm; 72%), whereas the high-arid climate stage was rich in accessions with dark colored spikes (72%) and black awns (96%). No particular phenotypic class was observed within the low semi-arid climate (Table S9).

#### Analysis of genetic diversity among different regions and climate stages

The results of ANOVA showed that 19 and 10% of the total genetic diversity was observed among regions and among climate stages, respectively, while 81 and 90% of the genetic variability was explained by differences within regions and within climate stages, respectively (Table 4).

Genetic diversity among regions showed Ne ranging from 1.366 (Sousse) to 3.031 (Gabes) (Table 3). Overall and among all investigated regions, Sousse showed the lowest genetic diversity indexes, while Gabes showed the highest genetic diversity indexes; the number of MLGs was the highest in Gabes (31) and lowest in Sousse and Medenine (7), and the Shannon’s information index was also the highest in Gabes (*H′* = 1.296) and lowest in Sousse (*H′* = 0.305). Moreover, the percentage of polymorphic loci (*P*) was 100% for all regions, except Sousse (50%). Moreover, the number of private alleles was also the highest in Gabes (PA = 17) and lowest in Sousse and Medenine (PA = 1). The value of *Fis* was greater than 0.800 in each region, except Sousse (*Fis* = 0.691). Interestingly, the DA number and heterozygosity index were the highest in Sousse. In fact, three diagnostic alleles (frequency > 70%; *Ho* = 0.100) were registered in Sousse, whereas only one such allele was identified in Gabes.

Analysis of SSR data obtained from different climate stages revealed that the mid-arid climate was the most outstanding, with the highest number of effective alleles (Ne = 3.174), the highest Shannon’s information index (*I* = 1.318) and the highest number of private alleles (PA = 19). By contrast, the high-arid climate stage showed the lowest number of effective alleles (Ne = 2.707), the lowest Shannon’s information index (*I* = 1.050) and the lowest number of private alleles (PA = 2). However, the value of *Fis* was similar (> 0.800) among all studied climate stages (Table 3).

#### Correlation between genetic distance and geographic distance

**Fig. 4:**
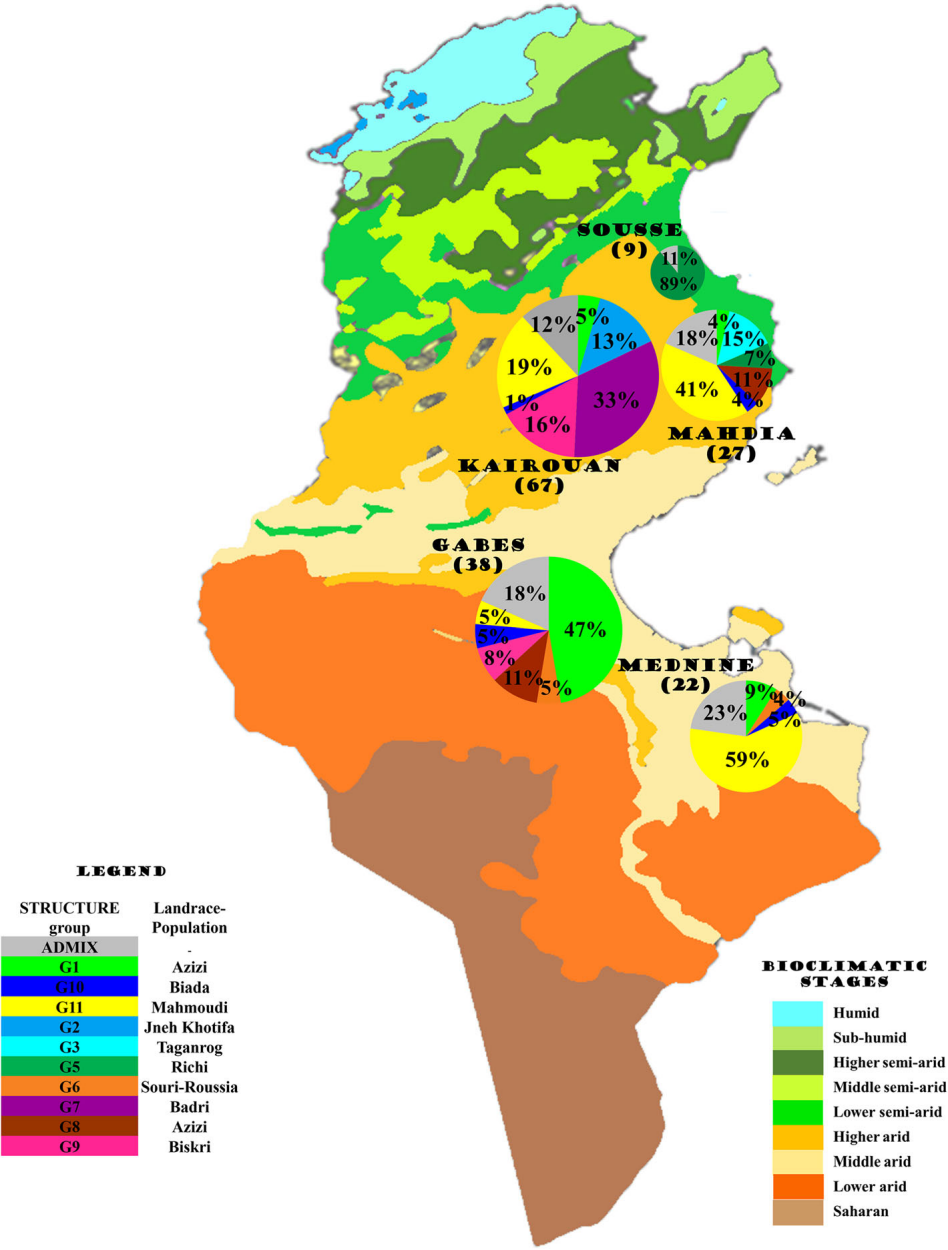
Geographic distribution of the 11 genetic groups (G1-G11), defined by STRUCTURE (version 2.3.4) [26] on 163 geo-localized durum wheat accessions genotyped with 10 SSR markers, over the regions of origin and the bioclimatic stages in Tunisia (https://www.d-maps.com/)

The Mantel test showed a significant correlation (*P* = 0.010; R_xy_ = 0.286) between genetic and geographic distances among durum wheat accessions, suggesting that accessions growing in close geographical proximity were genetically related. Azizi and Mahmoudi landraces showed the most widespread geographical distribution in central and southern Tunisia, except Sousse, and all climate stages. However, Azizi was more frequent in Gabes (25 accessions out of 38), while Mahmoudi was mostly found in Medenine (13 accessions out of 22) and Mahdia (11 accessions out of 27) (Fig. 4). In addition, all G5 genotypes, corresponding to the Richi landrace, were found in Sousse; all G7 and G2 genotypes, corresponding to Badri and Jneh Khotifa landraces, respectively, were found in Kairouan; and the landrace Taganrog, representative of the genetic group G3, was exclusively found in Mahdia.

Furthermore, we compared morphological traits between Azizi and Mahmoudi accessions collected from central and southern Tunisia. None of the traits, showed significant differences (*P* > 0.05), except for spike density (SD) which showed significant differences within Mahmoudi (*P* = 0.00). Mahmoudi accessions collected from central Tunisia had compact spikes (SD = 7), whereas those collected from southern Tunisia were characterized by loose spikes (SD = 5) (Table 5).

## Discussion

### Genetic and morphological diversity within the Tunisian durum wheat germplasm

In the present study, we investigated the genetic diversity of 302 Tunisian durum wheat accessions using 10 SSR markers, which enabled maximal differentiation among MLGs, suggesting that these markers have a good resolution power. Overall, the studied collection was characterized by high genetic diversity (overall Na = 9.9; PIC = 0.690; *He* = 0.346). Similar level of polymorphism (Na = 8; PIC = 0.68) was previously reported using 15 SSR markers in a Tunisian durum wheat collection composed of 7 modern cultivars and 27 old cultivars [10]. More recently, Slim et al. [22] reported genetic diversity indexes (PIC = 0.57; *He*: 0.28–0.82; Na: 2–13) of Tunisian durum wheat germplasm, consisting of 41 traditional varieties and 13 cultivars, using 16 SSR markers. A higher level of polymorphism (Na = 10; *He* = 0.71) was reported in a wider geographical collection of 172 durum wheat landraces (collected from 21 Mediterranean countries) and 20 modern cultivars genotyped by 44 SSR markers [27]. However, lower genetic diversity was observed in 33 Anatolian, 136 south Italian and 40 North-West African durum wheat landraces using 14, 44 and 29 SSR markers, respectively [7, 15, 28]. Robbana et al. [21] also reported low genetic diversity (PIC = 0.1; *He* = 0.25) in 196 Tunisian durum wheat accessions; this was possibly due to (i) the use of bi-allelic DArTseq markers, which are less informative than the multi-allelic SSR markers, and (ii) to limited number of landraces (5). This variability between studies suggests that the ability to capture the maximum genetic diversity depends on the type of markers, number of landraces, their origin and geographical distribution.

In this study, the level of phenotypic diversity detected on the basis of 12 morphological traits was consistent with that of genetic diversity, with a Shannon-Weaver index (*H′*) of 0.80. The morphological diversity observed in this study was higher than that described previously for 930 Tunisian durum wheat accessions (*H′* = 0.53), collected from fewer sites in southern Tunisia, using 22 qualitative and 3 quantitative traits [19]. Lower phenotypic diversity was also observed for Moroccan durum wheat populations composed of 101 landraces (*H′* = 0.62) [29] and 59 traditional durum wheat accessions (*H′* = 0.78) [30] using nine agro-morphological traits. Ethiopian durum wheat populations composed of 32 landraces showed an *H′* value of 0.71 using eight qualitative traits [31], while Oman populations composed of 161 accessions showed *H′* value of 0.52 and 0.66 using 15 qualitative and 17 quantitative traits, respectively [32].

In this study, SL (*H′* = 0.98), GSz (*H′* = 0.94), GSp (*H′* = 0.87), GC (*H′* = 0.86) and SS (*H′* = 0.86) were the most polymorphic morphological traits. Previous studies on Tunisian durum wheat populations showed different results for polymorphic traits, based on UPOV and IPGRI. Belhadj et al. [19] concluded that the most polymorphic traits were width of the truncation (*H′* = 0.97) and spike color (*H′* = 0.92), whereas Ayed et al. [20] revealed that grain number per spike (*H′* = 0.91), yield (*H′* = 0.89), plant height (*H′* = 0.87) and thousand kernel weight (*H′* = 0.86) showed the highest diversity index values. Slim et al. [33] reported that high polymorphism for awn anthocyanin pigmentation (*H′* = 1.18), spike glaucosity (*H′* = 0.89), hairiness on the external surface (*H′* = 0.88), awn color (*H′* = 0.78) and awn length relative to spike length (*H′* = 0.77). Ayed and Slim [34] revealed that spike density (*H′* = 0.86), glume pubescence (*H′* = 0.80) and glume color (*H′* = 0.79) showed the highest diversity index values. These differences among studies were essentially related to landraces. Indeed, Ayed et al. [20] assessed 17 Tunisian durum wheat landraces, which may have contributed to the wider range of morphological variation, whereas the present study and other studies evaluated fewer landraces. Thus, increasing the number of landraces would allow capturing a greater agro-morphological diversity.

### Population structure, network analysis and relationships with morphological characterization

In this study, the Tunisian durum wheat germplasm collection was genetically structured into 11 groups, which were consistent with morphological characteristics. Indeed, 8 out of 11 landraces corresponded to distinct genetic groups. This result highlights the effectiveness of SSR markers in distinguishing wheat varieties; thus, these markers continued to be widely exploited for DNA fingerprinting in plants [35]. DArTseq markers also grouped the Tunisian old durum wheat accessions according to the corresponding landrace nomenclature [21], confirming that genetic structure is modulated by farmer-directed selection pressure. Moreover, SSR markers divided Azizi accessions into two different genetic groups, which were initially collectively considered as a single landrace. However, 10 SSR markers were not sufficient to differentiate accessions of Souri and Roussia, as these were clustered together in a single genetic group. Therefore, we speculate that increasing the number of SSR markers would most likely improve the genetic differentiation between these landraces. Identification of 11 landraces, based on the spike and grain characteristics and in accordance with SSR fingerprinting data, suggests that morphological characterization is an efficient tool for varietal discrimination. Based on a landrace collection named by farmers, Mahmoudi and Biskri landraces were grouped into a single genetic group, as described by Robbana et al. [21]. This result highlights the importance of landrace identification based on a precise characterization of spike related traits and not solely on farmer-determined nomenclature. In fact, mixtures of different spike morphologies are often observed in a single field [19]. In addition, the mixture of landraces favors hybridization between different genetic groups, which explains the observed level of admixture (14%) herein. Most of the genetic groups displayed high level of genetic diversity, which is related to the high frequency of different MLGs. A predominance of a single MLG was found in Badri (G3) and Mahmoudi (G11), thereby reducing their genetic diversity. A predominant MLG of Mahmoudi landrace can be explained by the selection and multiplication, since 1908–1909, of a high-yielding accession aimed at increasing farmer income. In fact, the Mahmoudi landrace is preferred for its straw and grain yield as well as its ability to produce a high yield under drought and heat stress conditions prevalent in southern Tunisia. By contrast, Karim is the most popular modern variety in northern and central Tunisia, where heat and drought are not major problems. Badri is an old cultivar (released in 1969) obtained from a cross between two old landraces, Zenati Bouteille and Mahmoudi [11, 13]. Therefore, the predominant MLG of Badri would correspond to previously released lines.

A high gene flow was observed between regions (*Nm* = 1.037) and climate stages (*Nm* = 3.813). In fact, Mahmoudi (G11), Beskri (G9) and Azizi (G1 and G8), together accounted for 47% of the entire durum wheat collection, were distributed across different geographical locations. The widespread distribution of these landraces is explained by their earlier introduction (since 1893 or 1894) from Algeria and southern Europe, followed by their spread through local seed commercial trade and seed exchange between farmers. Thus, the exchange of seeds of different landraces between farmers from distant regions, followed by the introgression of these landraces into the pre-existing germplasm, could explain the level of high genetic variation observed within regions (81%) and climate stages (90%). Gabes and Mahdia showed the highest diversity indexes (including the number of MLGs), where 80 and 77% of the accessions, respectively had a unique MLG; and the highest number of private alleles (at a frequency < 0.4). In Gabes, 72% of the accessions belonged to cluster C1, whereas in Mahdia, 22 and 60% of the accessions belonged to clusters C1 and C2, respectively. This result suggests that a more diverse germplasm resource was available to farmers in Mahdia than to farmers in Gabes for breeding purposes. In fact, in Mahdia, 27 accessions were identified as belonging to five different landraces with a frequency less than 41%, whereas, 38 accessions were described in Gabes, with a predominance of the Azizi landrace (47%). Moreover, a high morphological diversity was observed among regions (*H′* = 0.55) due to the presence of different phenotypic classes for all the studied phenotypic traits within regions. According to Chentoufi et al. [30], the presence of wide morphological variability in wheat in different traditional agroecosystems could be explained by different seed exchange practices between farmers from different regions. Indeed, traditional management systems contributed effectively to the conservation of diversity of local durum wheat populations [36, 37] and the maintenance of landrace varietal characteristics in Tunisia. Nevertheless, gene flow could be counter-balanced by farmer selection for preferred landraces, which could result in locally adapted accessions. In fact, a moderate genetic variability (19%) was observed among regions. This ascertainment highlights the effect of selection pressure directed by farmers, based on their preferences for specific wheat types in the preparation of local traditional dishes, which may have led to the adaptation and predominance of landraces in certain eco-geographical zones [7, 22, 28, 30, 38, 39]. Notably, Kairouan, Sousse and Mahdia were characterized by specific landraces. For example, landraces Badri and Jneh Khotifa were only found in Kairouan, whereas landraces Taganrog and Richi were only found in Mahdia and Sousse, respectively. In addition, distinct phenotypic classes were detected within regions and climate stages (frequency > 70%), indicating that these classes might be characteristic of certain geographical areas, and environmental conditions may play a role in shaping the phenotypic diversity of durum wheat landraces. A local genetic adaptation pattern was also revealed in this study. For example, Gabes and Sousse were characterized by the presence of diagnostic alleles. Farmers in these regions have been selecting seeds and cultivating their old traditional landraces over many generations. This practice would result in the local adaptation of germplasm for a given eco-geographical environment [22]. Landraces under cultivation might undergo evolutionary changes if farmers keep using their own seed stock [38]. In fact, Fayaz et al. [40] defined landraces as locally adapted genotypes resulting from different environmental conditions and agricultural practices among ancient communities.

In addition to farmer’s selection pressure, natural selection was found to affect morphological characteristics within a single landrace, Mahmoudi. Mahmoudi accessions collected from southern Tunisia showed significantly looser spikes than those collected from central Tunisia (compact spikes). We speculate that the loose spike, characterized by an open glume in southern Tunisian Mahmoudi accessions, could provide tolerance to high temperature by maintaining fertility, as shown in rice germplasm [41]. The loose spike trait of southern Tunisian Mahmoudi accessions could be used in breeding programs for developing heat stress tolerance, and for the identification of genes and mechanisms involved in flower development useful, thus improving wheat adaptation to arid and marginal environments.

MSN analysis grouped the accessions into two major clusters, C1 and C2. However, neither one of these clusters correlated with the geographical origin of landraces. Notably, both Mahmoudi and Biskri were introduced in Tunisia from Algeria, while Jneh Khotifa, Azizi, Mekki, Biada and Roussia were considered local landraces cultivated mainly in northern and central Tunisia. Although various origins have been reported for landraces Azizi and Mekki, no origin has been reported for Richi and Taganrog, which are very old, but non-local, landraces [12, 13]. According to Deghais et al. [13], the landrace Jneh Khotifa was also known as Jneh Zarzoura and/or Kahla; the denomination of landrace Souri was extended in 1915 to Sarebouza obtained from Armenia. Soriano et al. [27], used 44 SSRs to show that Tunisian durum wheat landraces have four geographical origins, including East Mediterranean, East Balkan and Turkey, West Balkan and Egypt, and West Mediterranean, with dominance (> 50%) of the West Mediterranean genetic group. In addition, Sorriano et al. [27] demonstrated that western Mediterranean landraces are characterized by the heaviest grain weight compared with the other three genetic groups. In the current study, grain size did not significantly differ between C1 and C2 accessions, suggesting that both clusters contain accessions of the western Mediterranean origin. Moreover, Robbana et al. [21] reported that most of Tunisian landraces were introduced from the early Carthage trade maritime activity in the Mediterranean Sea through Lebanon, Greece and Italy.

## Conclusions

Tunisian old durum wheat, characterized here by both high genetic and morphological diversity, represents an important and valuable genetic resource that should be included in breeding and well-established conservation programs. In this study, we showed that Tunisian old durum wheat is structured into landraces, revealing the effect of selection pressure directed by farmers for specific wheat types and agro-morphologies. Nevertheless, the morpho-geographical spike density trait, apparent specifically in Mahmoudi accessions, suggests that environmental selection also acted on Tunisian durum wheat. Thus, our results provide important data for improving the adaptation of wheat to extreme or fluctuating Mediterranean conditions. Further physiological and agronomic analyses are needed to ascertain whether the spike density trait could be exploited in durum wheat breeding programs for tolerance to heat and drought.

## Methods

### Collection and multiplication of local durum wheat accessions

A collection of 304 old durum wheat accessions provided by the National Gene Bank of Tunisia (BNG) were used in this study. Accessions were collected from five regions in three distinct climate stages: Sousse and Mahdia (low semi-arid climate) and Kairouan (high-arid climate) located in central Tunisia, and Gabes and Medenine (mid-arid climate) located in southern Tunisia. The Global Positioning System (GPS) coordinates of 163 out of 304 accessions were registered (Table S10). Seeds of each accession were sown and increased from a single spike-derived lineage by the BNG team, and a BNG code was assigned to each accession. All accessions were further multiplied for spike characterization. All accessions used in this study have been preserved at the BNG of Tunisia and are available upon request.

### DNA extraction and SSR marker-based genotyping

Five seeds collected from one spike of each accession were germinated and grown under controlled conditions (20 °C day/16 °C night temperature, 16-h light/8-h dark photoperiod and 70% relative humidity) at Bioger research unit, INRAE, France. At the seedling stage (Zadok scale: 13–14), one leaf of each accession was sampled and placed in an extraction plate. The plates were placed at − 80 °C for 12 h before DNA extraction. DNA was extracted from the leaf samples of each of the 304 accessions using the DNeasy PowerPlant Pro HTP 96 Kit (Qiagen, France). DNA concentrations were quantified using a Nanodrop spectrophotometer (ND-1000) and stored at − 20 °C until needed for subsequent processing. The DNA of each accession was adjusted to a concentration of 15 ng·μl^− 1^ and genotyped using 10 SSR markers (Table S4), which were selected from a collection of 15 SSR markers used previously [29]. Forward primers were labeled with fluorescent dyes, and SSR markers were multiplexed, as described by Gautier et al. [42]. Each multiplex PCR, followed by gel electrophoresis, was performed according to the protocol established by Eurofin (https://www.eurofins.fr). Briefly, PCR was performed by preheating the DNA at 95 °C for 5 min, followed by 35 cycles of 95 °C for 30 s, 60 °C for 90 s and 72 °C for 30 s, with a final extension step of 60 °C for 30 min. PCR products were analyzed by electrophoresis on a 2% agarose gel, and fragments were separated according to their size on an ABI Prism Genetic Analyzer (Applied Biosystems). Data was checked again using the Peak scanner software (version 1.0) (https://www.thermofisher.com). Two accessions with missing data for all SSRs were excluded from the study.

### Morphological characterization of durum wheat accessions

Morphological characterization was carried out using five spikes per accession (total 1520 spikes). Accessions were evaluated using 12 quantitative and qualitative spike- and grain-related morphological traits. Spike evaluation was based on durum wheat descriptor standards of the IPGRI [23] and UPOV [24] (Table S11). Spike and grain morphological traits, defined by distinct phenotypic classes, were visually and numerically estimated. Traits including SC, GlC, AC, GC, SD, SS, GSp, GSz, and AL relative to SL were visually assessed, whereas other traits such as GSz, SL, NS and GN were quantified and then converted into codes. Subsequently, all accessions were named based on the catalog of cereal varieties cultivated in Tunisia [13]. This catalog serves as a reference for reporting and describing typical varietal characteristics of more than 40 old local durum wheat landraces cultivated in Tunisia.

### Data analysis

#### Polymorphism of SSR markers

Based on the SSR data generated on 302 accessions, the number of MLGs were identified with using the GIMLET software (version 1.3.2) [43]. To check the resolution of the 10 SSR markers used in this study, a genotype accumulation curve, was generated under R software [44] using the package ‘*pegas*’ package and *‘genotype_curve*’ function in the R 3.3.2 [44]. This analysis determines the minimum number of loci necessary to discriminate between the Tunisian durum wheat genotypes by randomly sampling up to *n*^*− 1*^ loci (without replacement) and counting the number of MLGs observed.

To assess the informativeness of SSR markers, the average PIC value of each marker was calculated by determining the frequency of alleles per locus using the following equation [45]:

$$ \mathbf{PIC}=1-\sum \limits_{i=1}^n{f}_i^2 $$

where *fi* is the frequency of the *i*^*th*^ allele in the set of 302 genotypes. SSR markers with PIC ≥0.5 were considered informative.

#### Polymorphism of morphological traits

Frequencies of different phenotypic classes were calculated for each of the 12 spike- and grain-related traits in the entire collection, by landraces (Table S1), regions and climate stages (Table S9). Based on these frequencies, the Shannon-Weaver index (*H′*) was calculated for each trait using the Past software [46]. *H* was estimated for the entire durum wheat collection, for accessions in different regions and climate stages and for each landrace. Each value of *H* was standardized by conversion to a relative phenotypic diversity index (*H′*) to express the values of *H′* within the range of 0–1. The Shannon-Weaver index (*H′*) was calculated as follows:

$$ H\hbox{'}=H/{H}_{\mathrm{max}} $$

where *H*_max_ Ln (S), S = the number of phenotypic classes.

#### Morphological and genetic structures

To investigate the morphological structure of the 304 accessions, PCA was performed based on 12 spike and grain related traits using R 3.3.2 [44]. The population genetic structure of these accessions was analyzed based on MLGs using STRUCTURE software (version 2.3.4) [26]. The STRUCTURE program was run with K values ranging from 1 to 20 in an admixture ancestry model by applying 10 independent runs for each K value, 100,000 burn-in phase iterations and 100,000 Markov Chain Monte Carlo (MCMC) iterations. The run with maximum likelihood was used to assign individual genotypes to different genetic groups. Genotypes with affiliation probabilities (inferred ancestry) > 75% were assigned to a distinct genetic group, and those with inferred ancestry < 75% were treated as admixed. The optimal number of genetic groups was determined using the mean posterior probability (ln P(D)) value per cluster (K) and the delta-K method of ln P(D) STRUCTURE harvester (version 0.6.94) [47].

To classify the 302 accessions according to their genetic relationship, MSN analysis was conducted based on Bruvo’s distance [48] using ‘*poppr*’ and ‘*adegenet*’ packages in R 3.3.2 [44]. Furthermore, the average value of each of the 12 traits was calculated for accessions belonging to the different clusters, as defined by the MSN analysis, using the following equation:

$$ \mathrm{Mean}={\sum}_{i=1}\left(n{C}_i\right)/\mathrm{N} $$

where N is the number of genotypes per genetic cluster, as defined by the MSN analysis; *n* is the number of individuals per phenotypic class; and C_i_ is the *i*^*th*^ phenotypic class per morphological trait.

To determine significant differences between genetic clusters for each morphological trait, ANOVA of calculated means was carried out using R 3.3.2 [44].

#### Analysis of population structure based on different regions and climate stages

Values of Na, Ne, PA (alleles specific to a single population), *I*, *He*, *Ho*, *Fis*, *P*, and DA (rare alleles with frequency > 70% for a genetic group or region and < 30% for others) were calculated within each genetic group, region and climate stage using GenAlEx (version 6.501) [25]. In addition, the correlation between genetic distance and log-transformed geographic distance (1 + geographic distance) of accessions was analyzed using a Mantel test [49] for the entire collection with GenAlEx (version 6.501) [25]. Correlations between the genetic distance matrix and morphological distance matrix were also assessed using a Mantel test. Furthermore, AMOVA was performed using GenAlEx (version 6.501) [25] to investigate the significance of genetic group differentiation (as defined by STRUCTURE) and genetic variability explained by regions and climate stages.

Moreover, mean values of all 12 spike and grain related traits were estimated for Azizi and Mahmoudi accessions located in different climate stages of central and southern Tunisia. To test potential regional effects on morphological traits, ANOVA of mean values was conducted in R 3.3.2 [44].

## Supplementary Information

**Additional file 1: Table S1.** Frequencies of the different phenotypic classes calculated for each trait by landraces.

**Additional file 2: Table S2.** Main morphological characteristics of 11 landraces identified across 304 Tunisian durum wheat accessions based on IPGRI (1985) [23], UPOV (1988) [24] and Deghais et al. [13].

**Additional file 3: Table S3.** Shannon-Weaver index (*H′*) estimated on the 11 Tunisian durum wheat landraces.

**Additional file 4: Table S4.** List of Single Sequence Repeat (SSR) markers with their chromosome allocation, forward and reverse primer sequences, size and dye used.

**Additional file 5: Figure S1.** Genotype accumulation curve generated under R 3.3.2 [44], for the Tunisian durum wheat landraces accessions genotyped with 10 SSR markers.

**Additional file 6: Table S5.** Summary of Private Alleles.

**Additional file 7: Table S6.** Pairwise Nei’s genetic distances between the durum wheat genetic groups based on 10 SSR markers.

**Additional file 8: Table S7.** Shannon-Weaver index (*H′*) estimated for the genetic clusters C1 and C2 defined by MSN analysis.

**Additional file 9: Table S8.** Diversity indexes of the genetic clusters C1 and C2 defined by MSN analysis.

**Additional file 10: Table S9.** Frequencies of the different phenotypic classes calculated for each trait by regions and by climatic stages.

**Additional file 11: Table S10.** Global Positioning System coordinates, number of durum wheat accessions and regions of the different collecting sites provided by The National Gene Bank.

**Additional file 12: Table S11.** Descriptors used for estimating spike- and grain-based trait diversity in the durum wheat landraces.

## Data Availability

The data sets supporting the results of this article are included in this manuscript and its additional information files.

## Abbreviations

AMOVA: Analysis of molecular variance
C: Genetic cluster as defined by MSN analysis
G: Genetic group as defined by STRUCTURE
*H′*: Shannon-Weaver index
MSN: Minimum spanning network
PCA: Principal component analysis
PIC: Polymorphic information content
