# Supplementary material for: Morphological characterization and genetic diversity analysis of Tunisian durum wheat (Triticum turgidum var. durum) accessions
Source: BMC Genom Data. 2021 Feb 3;22:3. doi: 10.1186/s12863-021-00958-3 (PMC7860204; doi:10.1186/s12863-021-00958-3)
Supplement: Supplementary file 1 — Additional file 1: Table S1. Frequencies of the different phenotypic classes calculated for each trait by landraces. [file 12863_2021_958_MOESM1_ESM.docx]

**Table S1.** Frequencies of the different phenotypic classes calculated for the 12 morphological traits by landraces*

| **Landraces** | **N** | **SC** | **SS** | **SD** | **SL** | **AL** | **AC** | **NS** | **GlC** | **GC** | **GSp** | **GSz** | **GN** |
| --- | --- | --- | --- | --- | --- | --- | --- | --- | --- | --- | --- | --- | --- |
| **Azizi**  **(G1/G8)** | 42 | 0.00 | 0.00 | 0.67 | 0.00 | 0.00 | 0.00 | 0.00 | 0.00 | 0.00 | 0.00 | 0.00 | 0.00 |
|  |  | 1.00 | 0.00 | 0.33 | 1.00 | 0.02 | 0.02 | 0.48 | 0.98 | 0.00 | 1.00 | 0.98 | 1.00 |
|  |  | 0.00 | 0.00 | 0.00 | 0.00 | 0.98 | 0.33 | 0.52 | 0.02 | 0.02 | 0.00 | 0.02 | 0.00 |
|  |  |  | 1.00 | 0.00 |  |  | 0.64 |  |  | 0.98 |  |  |  |
|  |  |  | 0.00 |  |  |  |  |  |  |  |  |  |  |
| **Jneh Khotifa**  **(G2)** | 12 | 0.00 | 0.00 | 0.00 | 0.00 | 0.00 | 0.00 | 0.00 | 0.00 | 0.00 | 0.00 | 0.00 | 0.00 |
|  |  | 0.00 | 1.00 | 0.00 | 0.00 | 0.00 | 0.00 | 0.00 | 0.00 | 0.00 | 1.00 | 0.00 | 0.00 |
|  |  | 1.00 | 0.00 | 0.00 | 1.00 | 1.00 | 0.00 | 1.00 | 1.00 | 0.00 | 0.00 | 1.00 | 1.00 |
|  |  |  | 0.00 | 1.00 |  |  | 1.00 |  |  | 1.00 |  |  |  |
|  |  |  | 0.00 |  |  |  |  |  |  |  |  |  |  |
| **Taganrog** | 27 | 1.00 | 0.00 | 0.00 | 0.15 | 0.00 | 0.00 | 0.00 | 0.04 | 0.37 | 0.00 | 0.00 | 0.00 |
| **(G3)** |  | 0.00 | 0.00 | 1.00 | 0.85 | 0.00 | 0.00 | 0.00 | 0.00 | 0.00 | 1.00 | 0.81 | 0.00 |
|  |  | 0.00 | 0.00 | 0.00 | 0.00 | 1.00 | 0.00 | 1.00 | 0.96 | 0.63 | 0.00 | 0.19 | 1.00 |
|  |  |  | 1.00 | 0.00 |  |  | 1.00 |  |  | 0.00 |  |  |  |
|  |  |  | 0.00 |  |  |  |  |  |  |  |  |  |  |
| **Mekki** | 28 | 0.00 | 0.00 | 0.00 | 1.00 | 0.00 | 0.00 | 0.00 | 0.00 | 0.00 | 0.00 | 1.00 | 0.00 |
| **(G4)** |  | 1.00 | 1.00 | 0.00 | 0.00 | 0.00 | 0.11 | 0.36 | 1.00 | 0.00 | 1.00 | 0.00 | 1.00 |
|  |  | 0.00 | 0.00 | 0.00 | 0.00 | 1.00 | 0.14 | 0.64 | 0.00 | 1.00 | 0.00 | 0.00 | 0.00 |
|  |  |  | 0.00 | 1.00 |  |  | 0.75 |  |  | 0.00 |  |  |  |
|  |  |  | 0.00 |  |  |  |  |  |  |  |  |  |  |
| **Richi** | 11 | 1.00 | 1.00 | 0.00 | 0.00 | 0.00 | 1.00 | 0.00 | 1.00 | 0.18 | 0.00 | 0.00 | 0.00 |
| **(G5)** |  | 0.00 | 0.00 | 0.00 | 0.18 | 0.82 | 0.00 | 0.18 | 0.00 | 0.00 | 0.00 | 0.18 | 0.18 |
|  |  | 0.00 | 0.00 | 0.09 | 0.82 | 0.18 | 0.00 | 0.82 | 0.00 | 0.82 | 1.00 | 0.82 | 0.82 |
|  |  |  | 0.00 | 0.91 |  |  | 0.00 |  |  | 0.00 |  |  |  |
|  |  |  | 0.00 |  |  |  |  |  |  |  |  |  |  |
| **Souri** | 10 | 0.00 | 0.00 | 1.00 | 0.00 | 1.00 | 0.00 | 0.00 | 0.00 | 0.00 | 1.00 | 0.00 | 0.00 |
| **(G6)** |  | 1.00 | 0.00 | 0.00 | 0.80 | 0.00 | 1.00 | 0.10 | 1.00 | 1.00 | 0.00 | 1.00 | 1.00 |
|  |  | 0.00 | 0.00 | 0.00 | 0.20 | 0.00 | 0.00 | 0.90 | 0.00 | 0.00 | 0.00 | 0.00 | 0.00 |
|  |  |  | 1.00 | 0.00 |  |  | 0.00 |  |  | 0.00 |  |  |  |
|  |  |  | 0.00 |  |  |  |  |  |  |  |  |  |  |
| **Roussia** | 7 | 0.00 | 0.00 | 1.00 | 0.00 | 0.00 | 0.00 | 0.14 | 0.00 | 0.00 | 1.00 | 0.00 | 1.00 |
| **(G6)** |  | 1.00 | 0.00 | 0.00 | 0.29 | 0.00 | 1.00 | 0.14 | 1.00 | 1.00 | 0.00 | 1.00 | 0.00 |
|  |  | 0.00 | 1.00 | 0.00 | 0.71 | 1.00 | 0.00 | 0.71 | 0.00 | 0.00 | 0.00 | 0.00 | 0.00 |
|  |  |  | 0.00 | 0.00 |  |  | 0.00 |  |  | 0.00 |  |  |  |
|  |  |  | 0.00 |  |  |  |  |  |  |  |  |  |  |
| **Badri** | 22 | 1.00 | 0.00 | 0.00 | 1.00 | 0.00 | 0.00 | 1.00 | 0.00 | 1.00 | 1.00 | 1.00 | 0.00 |
| **(G7)** |  | 0.00 | 0.00 | 0.00 | 0.00 | 0.00 | 0.00 | 0.00 | 0.00 | 0.00 | 0.00 | 0.00 | 1.00 |
|  |  | 0.00 | 0.00 | 1.00 | 0.00 | 1.00 | 0.00 | 0.00 | 1.00 | 0.00 | 0.00 | 0.00 | 0.00 |
|  |  |  | 1.00 | 0.00 |  |  | 1.00 |  |  | 0.00 |  |  |  |
|  |  |  | 0.00 |  |  |  |  |  |  |  |  |  |  |
| **Biskri** | 46 | 1.00 | 0.00 | 0.00 | 0.00 | 0.00 | 0.00 | 0.00 | 1.00 | 0.00 | 0.00 | 0.00 | 0.00 |
| **(G9)** |  | 0.00 | 0.24 | 0.24 | 0.00 | 1.00 | 0.00 | 0.00 | 0.00 | 0.00 | 0.80 | 0.07 | 0.22 |
|  |  | 0.00 | 0.00 | 0.76 | 1.00 | 0.00 | 0.02 | 1.00 | 0.00 | 1.00 | 0.20 | 0.93 | 0.78 |
|  |  |  | 0.00 | 0.00 |  |  | 0.98 |  |  | 0.00 |  |  |  |
|  |  |  | 0.76 |  |  |  |  |  |  |  |  |  |  |
| **Biada** | 24 | 1.00 | 0.00 | 0.00 | 0.04 | 0.00 | 1.00 | 0.00 | 1.00 | 1.00 | 0.00 | 0.00 | 0.00 |
| **(G10)** |  | 0.00 | 0.00 | 1.00 | 0.96 | 1.00 | 0.00 | 1.00 | 0.00 | 0.00 | 1.00 | 1.00 | 0.71 |
|  |  | 0.00 | 0.00 | 0.00 | 0.00 | 0.00 | 0.00 | 0.00 | 0.00 | 0.00 | 0.00 | 0.00 | 0.29 |
|  |  |  | 1.00 | 0.00 |  |  | 0.00 |  |  | 0.00 |  |  |  |
|  |  |  | 0.00 |  |  |  |  |  |  |  |  |  |  |
| **Mahmoudi** | 74 | 1.00 | 1.00 | 0.00 | 0.28 | 0.00 | 0.18 | 0.00 | 0.86 | 1.00 | 0.00 | 0.00 | 0.00 |
| **(G11)** |  | 0.00 | 0.00 | 0.23 | 0.72 | 0.00 | 0.00 | 1.00 | 0.00 | 0.00 | 0.00 | 0.16 | 0.58 |
|  |  | 0.00 | 0.00 | 0.77 | 0.00 | 1.00 | 0.00 | 0.00 | 0.14 | 0.00 | 1.00 | 0.84 | 0.42 |
|  |  |  | 0.00 | 0.00 |  |  | 0.82 |  |  | 0.00 |  |  |  |
|  |  |  | 0.00 |  |  |  |  |  |  |  |  |  |  |
| **N:** Number of accessions per landrace**; SC :** spike color**; SS :** spike shape**; SD :** spike density**; SL :** spike length**; AL :** awn length**; AC :** awn color**; NS :** number of spikelets/spike**; GlC :** glume color**; GC :** grain color**; GSp :** grain shape**; GSz :** grain size **; GN :** number of grains/spikelet**; * Phenotypic classes per trait have the same order as mentioned in Table S11.** | | | | | | | | | | | | | |
