# Supplementary material for: Morphological characterization and genetic diversity analysis of Tunisian durum wheat (Triticum turgidum var. durum) accessions
Source: BMC Genom Data. 2021 Feb 3;22:3. doi: 10.1186/s12863-021-00958-3 (PMC7860204; doi:10.1186/s12863-021-00958-3)
Supplement: Supplementary file 2 — Additional file 2: Table S2. Main morphological characteristics of 11 landraces identified across 304 Tunisian durum wheat accessions based on IPGRI (1985) [23], UPOV (1988) [24] and Deghais et al. [13]. [file 12863_2021_958_MOESM2_ESM.docx]

**Table S2.** Main morphological characteristics of 11 landraces identified across 304 Tunisian durum wheat accessions based on IPGRI (1985) [23], UPOV (1988) [24] and Deghais et al. [13].

| **Landrace** | **12 studied Spike characteristics** | **Additional characteristics** |
| --- | --- | --- |
| **Azizi**  **(G1/G8)**  **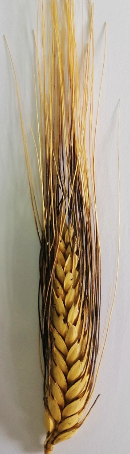** | **1. Spike color :** light tan  **2. Spike shape  :** rectangular  **3. Spike density :** lax to intermediate  **4. Spike length :** intermediate (6 to 9 cm)  **5. Number of spikelets /spike :** intermediate (16 to 22)  **6. Awn color :** Black  **7.** **Awn length :** longer than the spike  **8. Glume color :** tan with black spots  **9. Grain color :** brown  **10. Grain shape :** moderately long  **11. Grain size :** Intermediate (0.3 to 0.5 cm)  **12. Number of grains/spikelet :** intermediate (2 to 3) | **Glume Form :** long and tight  **Glume Hairiness :** absent  **Straw Color :** golden blond  **Plant Height :** intermediate to high (~120 cm) |
| **Jneh Khotifa**  **(G2)**  **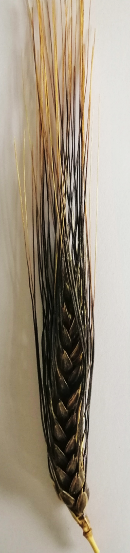** | **1. Spike color :** Black to purple  **2. Spike shape  :** elongate with parallel edges  **3. Spike density :** very dense  **4. Spike length :** long (>9 cm)  **5. Number of spikelets /spike :** high (>23)  **6. Awn color :** Black  **7. Awn length :** longer than the spike  **8. Glume color :** Black to purple  **9. Grain color :** dark brown  **10. Grain shape :** moderately long  **11. Grain size :** large (>0.5 cm)  **12. Number of grains/spikelet :** high (> 3) | **Glume Form :** short, sharp, curved inside  **Glume Hairiness :** high  **Straw Color :** blond  **Plant Height :** high (~150 cm) |
| **Taganrog**  **(G3)**  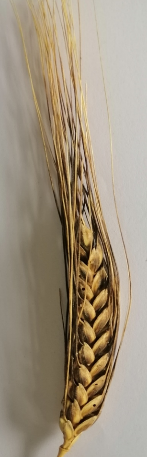 | **1. Spike color :** white  **2. Spike shape  :** flattened and half cylindrical  **3. Spike density :** intermediate  **4. Spike length :** short to intermediate  **5. Number of spikelets /spike :** high (>23)  **6. Awn color :** Black  **7. Awn length :** longer than the spike  **8. Glume color :** Black  **9. Grain color :** light brown  **10. Grain shape :** moderately long  **11. Grain size :** Intermediate (0.3 to 0.5 cm)  **12. Number of grains/spikelet :** high (> 3) | **Glume Form :** long and sharp, washed with black  **Glume Hairiness :** low  **Straw Color :** white  **Plant Height :** high (~120 to 140 cm) |
| **Mekki**  **(G4)**  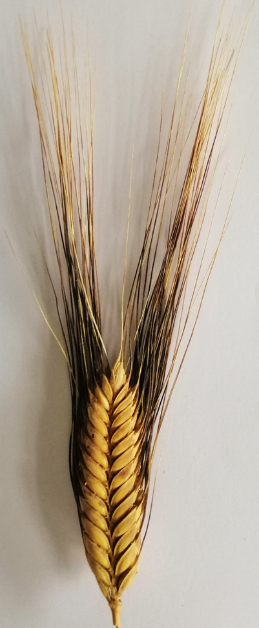 | **1. Spike color :** tan to red  **2. Spike shape  :** with parallel edges  **3. Spike density :** very dense  **4. Spike length :** short (< 6 cm)  **5. Number of spikelets /spike :** high (>23)  **6. Awn color :** Brown  **7. Awn length :** longer than the spike  **8. Glume color :** brown to red  **9. Grain color :** tan  **10. Grain shape :** moderately long  **11. Grain size :** small (<0.3 cm)  **12. Number of grains/spikelet :** intermediate (2 to 3) | **Glume Form :** short and curved  **Glume Hairiness :** absent  **Straw Color :** white  **Plant Height :** high (~120 cm) |
| **Richi**  **(G5)**  **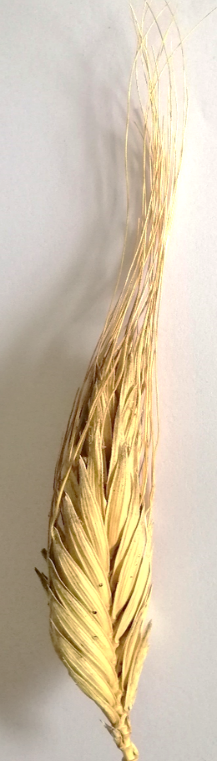** | **1. Spike color :** white  **2. Spike shape  :** stretched and pyramidal  **3. Spike density :** dense  **4. Spike length :** long (>9 cm)  **5. Number of spikelets /spike :** high (>23)  **6. Awn color :** White  **7. Awn length :** same length as the spike  **8. Glume color :** white to blond  **9. Grain color :** tan  **10. Grain shape :** elongate  **11. Grain size :** large (>0.5 cm)  **12. Number of grains/spikelet :** high (>3) | **Glume Form :** very long and feathery  **Glume Hairiness :** Presence of hair on Glume edges  **Straw Color :** white  **Plant Height :** high (~130 cm) |
| **Souri**  **(G6)**  **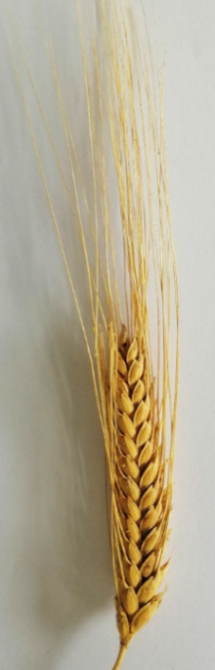** | **1. Spike color :** red  **2. Spike shape  :** rectangular, flattened, tight  **3. Spike density :** intermediate to lax  **4. Spike length :** intermediate to long (>9 cm)  **5. Number of spikelets /spike :** high (>23)  **6. Awn color :** Red  **7. Awn length :** shorter than the spike  **8. Glume color :** red  **9. Grain color :** yellow-orange  **10. Grain shape :** slightly long  **11. Grain size :** Intermediate (0.3 - 0.5 cm)  **12. Number of grains/spikelet :** intermediate (2 to 3) | **Glume Form :** moderately long  **Glume Hairiness :** absent  **Straw Color :** dark blond  **Plant Height :** intermediate (~100 to 110 cm) |
| **Roussia**  **(G6)**  **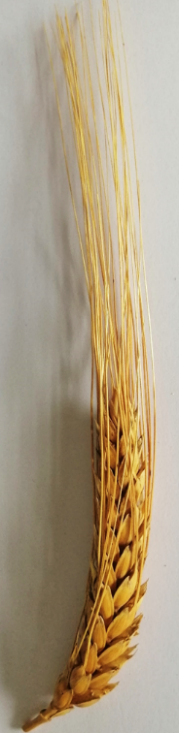** | **1. Spike color :** red  **2. Spike shape  :** long, cylindrical, very tight  **3. Spike density :** lax  **4. Spike length :** long (>9 cm)  **5. Number of spikelets /spike :** high (>23)  **6. Awn color :** Red  **7. Awn length :** longer than the spike  **8. Glume color :** red  **9. Grain color :** orange  **10. Grain shape :** slightly long  **11. Grain size :** Intermediate (0.3 to 0.5 cm)  **12. Number of grains/spikelet :** low (< 2) | **Glume Form :** horizontale  **Glume Hairiness :** absent  **Straw Color :** dark blond  **Plant Height :** intermediate (~110 cm) |
| **Badri**  **(G7)**  **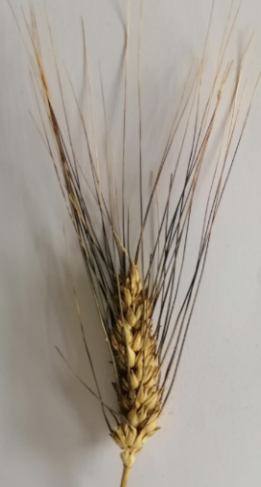** | **1. Spike color :** white greyish  **2. Spike shape  :** very short and large  **3. Spike density :** dense  **4. Spike length :** short (< 6 cm)  **5. Number of spikelets /spike :** low (<15)  **6. Awn color :** Black  **7. Awn length :** longer than the spike  **8. Glume color :** white with black bordure  **9. Grain color :** white  **10. Grain shape :** slightly long  **11. Grain size :** small (<0.3 cm)  **12. Number of grains/spikelet :** intermediate (2 to 3) | **Glume Form :** long  **Glume Hairiness :** absent  **Straw Color :** golden blond  **Plant Height :** short (~90 cm) |
| **Biskri**  **(G9)**  **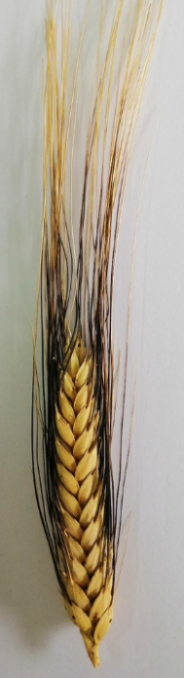** | **1. Spike color :** white  **2. Spike shape  :** fusiform and flattened  **3. Spike density :** intermediate to dense  **4. Spike length :** long (>9 cm)  **5. Number of spikelets /spike :** high (>23)  **6. Awn color :** Black  **7. Awn length :** longer than the spike  **8. Glume color :** white with a line on the sup edge  **9. Grain color :** dark tan  **10. Grain shape :** moderately long  **11. Grain size :** large (> 0.5 cm)  **12. Number of grains/spikelet :** high (>3) | **Glume Form :** long and very sharp with a black line on top  **Glume Hairiness :** low  **Straw Color :** white  **Plant Height :** high (~120 to 140 cm) |
| **Biada**  **(G10)**  **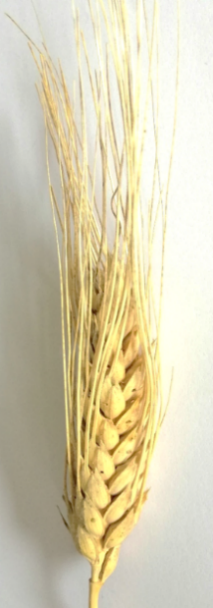** | **1. Spike color :** white  **2. Spike shape  :** round to half cylindrical  **3. Spike density :** intermediate to lax  **4. Spike length :** intermediate (6 to 9 cm)  **5. Number of spikelets /spike :** intermediate (16 to 22)  **6. Awn color :** White  **7. Awn length :** same length as the spike  **8. Glume color :** white with some spots  **9. Grain color :** white  **10. Grain shape :** moderately long  **11. Grain size :** Intermediate (0.3 to 0.5 cm)  **12. Number of grains/spikelet :** intermediate (2 to 3) | **Glume Form :** elongate  **Glume Hairiness :** very low  **Straw Color :** white  **Plant Height :** intermediate to short (~90 to 100 cm) |
| **Mahmoudi**  **(G11)**  **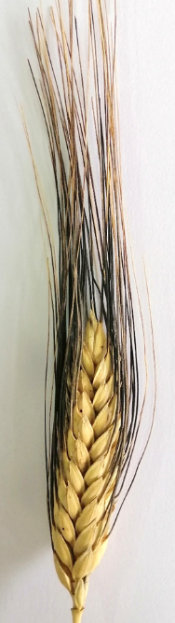** | **1. Spike color :** white to blond  **2. Spike shape  :** sub-pyramidal and flattened  **3. Spike density :** dense  **4. Spike length :** intermediate (6 to 9 cm)  **5. Number of spikelets /spike :** intermediate (16-22)  **6. Awn color :** Black  **7. Awn length :** longer than the spike  **8. Glume color :** white with black bordures  **9. Grain color :** white  **10. Grain shape :** long  **11. Grain size :** large (> 0.5 cm)  **12. Number of grains/spikelet :** high (>3) | **Glume Form :** short and oblique  **Glume Hairiness :** absent  **Straw Color :** blond  **Plant Height :** high (~120 to 130 cm) |
