# Supplementary material for: Morphological characterization and genetic diversity analysis of Tunisian durum wheat (Triticum turgidum var. durum) accessions
Source: BMC Genom Data. 2021 Feb 3;22:3. doi: 10.1186/s12863-021-00958-3 (PMC7860204; doi:10.1186/s12863-021-00958-3)
Supplement: Supplementary file 3 — Additional file 3: Table S3. Shannon-Weaver index (H′) estimated on the 11 Tunisian durum wheat landraces. [file 12863_2021_958_MOESM3_ESM.docx]

**Table S3.** Shannon-Weaver index (*H’*) estimated on the 11 Tunisian durum wheat landraces.

| **Landraces** | **SC** | **SS** | **SD** | **SL** | **AL** | **AC** | **NS** | **GlC** | **GC** | **GSp** | **GSz** | **GN** | **Mean *H'*** |
| --- | --- | --- | --- | --- | --- | --- | --- | --- | --- | --- | --- | --- | --- |
| **Azizi** | 0.00 | 0.00 | 0.46 | 0.00 | 0.10 | 0.53 | 0.63 | 0.10 | 0.08 | 0.00 | 0.10 | 0.00 | 0.17 |
| **Badri** | 0.00 | 0.00 | 0.00 | 0.00 | 0.00 | 0.00 | 0.00 | 0.00 | 0.00 | 0.00 | 0.00 | 0.00 | 0.00 |
| **Biada** | 0.00 | 0.00 | 0.00 | 0.16 | 0.00 | 0.00 | 0.00 | 0.00 | 0.00 | 0.00 | 0.00 | 0.55 | 0.06 |
| **Biskri** | 0.00 | 0.34 | 0.40 | 0.00 | 0.00 | 0.08 | 0.00 | 0.00 | 0.00 | 0.45 | 0.22 | 0.48 | 0.16 |
| **Jneh Khotifa** | 0.00 | 0.00 | 0.00 | 0.00 | 0.00 | 0.00 | 0.00 | 0.00 | 0.00 | 0.00 | 0.00 | 0.00 | 0.00 |
| **Mahmoudi** | 0.00 | 0.00 | 0.39 | 0.54 | 0.00 | 0.34 | 0.00 | 0.36 | 0.00 | 0.00 | 0.40 | 0.62 | 0.22 |
| **Mekki** | 0.00 | 0.00 | 0.00 | 0.00 | 0.00 | 0.53 | 0.59 | 0.00 | 0.00 | 0.00 | 0.00 | 0.00 | 0.09 |
| **Richi** | 0.00 | 0.00 | 0.22 | 0.43 | 0.43 | 0.00 | 0.43 | 0.00 | 0.34 | 0.00 | 0.43 | 0.43 | 0.23 |
| **Roussia** | 0.00 | 0.00 | 0.00 | 0.54 | 0.00 | 0.00 | 0.72 | 0.00 | 0.00 | 0.00 | 0.00 | 0.00 | 0.11 |
| **Souri** | 0.00 | 0.00 | 0.00 | 0.46 | 0.00 | 0.00 | 0.30 | 0.00 | 0.00 | 0.00 | 0.00 | 0.00 | 0.06 |
| **Taganrog** | 0.00 | 0.00 | 0.00 | 0.38 | 0.00 | 0.00 | 0.00 | 0.14 | 0.48 | 0.00 | 0.44 | 0.00 | 0.12 |
| **Mean** | 0.00 | 0.03 | 0.13 | 0.23 | 0.05 | 0.13 | 0.24 | 0.06 | 0.08 | 0.04 | 0.14 | 0.19 | **0.11** |

**SC :** spike color**; SS :** spike shape**; SD :** spike density**; SL :** spike length**; AL :** awn length**; AC :** awn color**; NS :** number of spikelets/spike**; GlC :** glume color**; GC :** grain color**; GSp :** grain shape**; GSz :** grain size **;**  **GN :** number of grains/spikelet
