# Supplementary material for: Morphological characterization and genetic diversity analysis of Tunisian durum wheat (Triticum turgidum var. durum) accessions
Source: BMC Genom Data. 2021 Feb 3;22:3. doi: 10.1186/s12863-021-00958-3 (PMC7860204; doi:10.1186/s12863-021-00958-3)
Supplement: Supplementary file 4 — Additional file 4: Table S4. List of Single Sequence Repeat (SSR) markers with their chromosome allocation, forward and reverse primer sequences, size and dye used. [file 12863_2021_958_MOESM4_ESM.docx]

**Table S4.** List of Single Sequence Repeat (SSR) markers with their chromosome allocation, forward and reverse primer sequences, size and Dye used.

| **Locus** | **Forward Primer** | **Reverse Primer** | **Chromosome Arm** | **Allele Size range (bp^1^)** | **Dye Eurofins** |
| --- | --- | --- | --- | --- | --- |
| **Xgwm413** | TGCTTGTCTAGATTGCTTGGG | GATCGTCTCGTCCTTGGCA | 2B | 90-110 | a |
| **Xgpw7148** | GCACACAACGACACTTGCTT | GCTTAGCTGCTTGCTTTGTG | 3B | 93-100 | a |
| **Xgwm495** | GAGAGCCTCGCGAAATATAGG | TGCTTCTGGTGTTCCTTCG | 4B | 157-191 | b |
| **Xgwm193** | CTTTGTGCACCTCTCTCTCC | AATTGTGTTGATGATTTGGGG | 6BS | 162-192 | b |
| **Xgpw2239** | CAACCATATGCCCAGGAGAC | TGTTGCTGTCTGAAACAGGG | 4AS | 191-193 | b |
| **Xgwm285** | ATGACCCTTCTGCCAAACAC | ATCGACCGGGATCTAGCC | 3B | 209-235 | c |
| **Xgpw4082** | CTTTCTTTCCCCTCCTGTCC | ATCATCACAAATGCAGCGAG | 4B | 213-228 | c |
| **Xgpw4004** | CGCCTCGGATTCTATTCTTG | CTTACTGCGGCCTTGAGTTG | 5A | 224-229 | c |
| **Xgpw2103** | CGTATGCAGCATGGCATC | GCTATGTTGTGTGGCATTGG | 7AL (7B) | 230-238 | c |
| **Xgwm372** | AATAGAGCCCTGGGACTGGG | GAAGGACGACATTCCACCTG | 2A | 312-314 | d |

**bp :** base pairs ; **Annealing temperature (TM)** = 60°C ; **a :** multiplex 1 (Ned-Fam) ; **b :** multiplex 2 (Fam-Ned-Vic) ; **c :** multiplex 3 (Ned-Fam-Pet-Vic) ; **d :** multiplex 4 (Fam)
