# Supplementary material for: Morphological characterization and genetic diversity analysis of Tunisian durum wheat (Triticum turgidum var. durum) accessions
Source: BMC Genom Data. 2021 Feb 3;22:3. doi: 10.1186/s12863-021-00958-3 (PMC7860204; doi:10.1186/s12863-021-00958-3)
Supplement: Supplementary file 5 — Additional file 5: Figure S1. Genotype accumulation curve generated under R 3.3.2 [44], for the Tunisian durum wheat landraces accessions genotyped with 10 SSR markers. [file 12863_2021_958_MOESM5_ESM.docx]

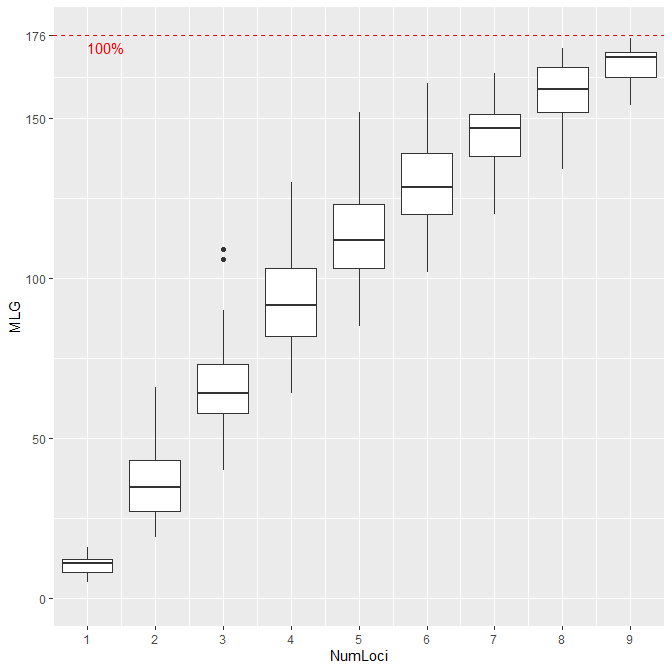


**Figure S1.** Genotype accumulation curve generated under R 3.3.2 [48], for the Tunisian durum wheat landraces accessions genotyped with 10 SSR markers.
