# Supplementary material for: Morphological characterization and genetic diversity analysis of Tunisian durum wheat (Triticum turgidum var. durum) accessions
Source: BMC Genom Data. 2021 Feb 3;22:3. doi: 10.1186/s12863-021-00958-3 (PMC7860204; doi:10.1186/s12863-021-00958-3)
Supplement: Supplementary file 6 — Additional file 6: Table S5. Summary of Private Alleles. [file 12863_2021_958_MOESM6_ESM.docx]

**Table S5.** Summary of Private Alleles.

|  |  | **Locus** | **Allele** | **Freq** |
| --- | --- | --- | --- | --- |
| **Subpopulations** | **ADMIX** | **L1** | 199 | 0.024 |
|  |  | **L2** | 194 | 0.049 |
|  |  | **L4** | 189 | 0.024 |
|  |  | **L6** | 228 | 0.024 |
|  |  | **L8** | 234 | 0.111 |
|  |  | **L10** | 305 | 0.014 |
|  | **G1** | **L1** | 101 | 0.229 |
|  |  | **L1** | 102 | 0.021 |
|  |  | **L2** | 186 | 0.083 |
|  | **G10** | **L10** | 312 | 0.063 |
|  | **G11** | **L10** | 299 | 0.017 |
|  | **G3** | **L8** | 286 | 0.053 |
|  | **G4** | **L10** | 323 | 0.154 |
|  | **G5** | **L4** | 175 | 0.200 |
|  |  | **L8** | 227 | 0.100 |
|  | **G6** | **L2** | 181 | 0.111 |
|  |  | **L6** | 213 | 0.111 |
|  |  | **L6** | 230 | 0.333 |
|  |  | **L6** | 232 | 0.111 |
|  |  | **L7** | 218 | 0.111 |
|  |  | **L8** | 222 | 0.111 |
|  |  | **L9** | 240 | 0.125 |
|  | **G8** | **L1** | 103 | 0.500 |
|  |  | **L1** | 104 | 0.100 |
|  |  | **L4** | 173 | 0.100 |
|  |  | **L4** | 177 | 0.100 |
|  | **G9** | **L8** | 235 | 0.250 |
|  |  | **L8** | 236 | 0.036 |
| **Regions** | **Gabes** | **L1** | 102 | 0.013 |
|  |  | **L1** | 104 | 0.026 |
|  |  | **L1** | 196 | 0.263 |
|  |  | **L1** | 199 | 0.026 |
|  |  | **L2** | 181 | 0.026 |
|  |  | **L2** | 186 | 0.053 |
|  |  | **L2** | 194 | 0.026 |
|  |  | **L4** | 177 | 0.026 |
|  |  | **L4** | 189 | 0.026 |
|  |  | **L6** | 213 | 0.026 |
|  |  | **L6** | 228 | 0.026 |
|  |  | **L7** | 216 | 0.026 |
|  |  | **L7** | 218 | 0.026 |
|  |  | **L8** | 234 | 0.026 |
|  |  | **L9** | 240 | 0.029 |
|  |  | **L10** | 305 | 0.014 |
|  |  | **L10** | 314 | 0.042 |
|  | **Kairouan** | **L1** | 192 | 0.060 |
|  |  | **L2** | 198 | 0.119 |
|  | **Mahdia** | **L1** | 100 | 0.037 |
|  |  | **L2** | 192 | 0.037 |
|  |  | **L3** | 172 | 0.037 |
|  |  | **L3** | 176 | 0.056 |
|  |  | **L4** | 173 | 0.037 |
|  |  | **L4** | 175 | 0.074 |
|  |  | **L4** | 187 | 0.037 |
|  |  | **L5** | 191 | 0.037 |
|  |  | **L8** | 227 | 0.040 |
|  |  | **L8** | 286 | 0.040 |
|  |  | **L10** | 299 | 0.045 |
|  | **Mednine** | **L8** | 222 | 0.045 |
|  | **Sousse** | **L3** | 174 | 0.111 |
| **Climatic stages** | **High-arid** | **L1** | 192 | 0.060 |
|  |  | **L2** | 198 | 0.119 |
|  | **Low semi-arid** | **L1** | 100 | 0.028 |
|  |  | **L2** | 192 | 0.028 |
|  |  | **L3** | 172 | 0.028 |
|  |  | **L3** | 174 | 0.028 |
|  |  | **L3** | 176 | 0.042 |
|  |  | **L4** | 173 | 0.028 |
|  |  | **L4** | 175 | 0.056 |
|  |  | **L4** | 187 | 0.028 |
|  |  | **L5** | 191 | 0.028 |
|  |  | **L8** | 227 | 0.029 |
|  |  | **L8** | 286 | 0.029 |
|  |  | **L10** | 299 | 0.032 |
|  | **Mid-arid** | **L1** | 102 | 0.008 |
|  |  | **L1** | 104 | 0.017 |
|  |  | **L1** | 196 | 0.167 |
|  |  | **L1** | 198 | 0.083 |
|  |  | **L1** | 199 | 0.017 |
|  |  | **L2** | 181 | 0.017 |
|  |  | **L2** | 186 | 0.033 |
|  |  | **L2** | 194 | 0.017 |
|  |  | **L4** | 177 | 0.017 |
|  |  | **L4** | 189 | 0.017 |
|  |  | **L6** | 213 | 0.017 |
|  |  | **L6** | 228 | 0.017 |
|  |  | **L7** | 216 | 0.017 |
|  |  | **L7** | 218 | 0.017 |
|  |  | **L8** | 222 | 0.017 |
|  |  | **L8** | 234 | 0.017 |
|  |  | **L9** | 240 | 0.018 |
|  |  | **L10** | 305 | 0.009 |
|  |  | **L10** | 314 | 0.026 |
| **L1**= Xgwm413 ; **L2**= Xgpw7148 ; **L3**= Xgwm495 ; **L4**= Xgwm193 ; **L5**= Xgpw2239 ; **L6**= Xgwm285 ; **L7**= Xgpw4082 ; **L8**= Xgpw4004 ; **L9**= Xgpw2103 ; **L10**= Xgwm372 | | | | |
