# Supplementary material for: Morphological characterization and genetic diversity analysis of Tunisian durum wheat (Triticum turgidum var. durum) accessions
Source: BMC Genom Data. 2021 Feb 3;22:3. doi: 10.1186/s12863-021-00958-3 (PMC7860204; doi:10.1186/s12863-021-00958-3)
Supplement: Supplementary file 7 — Additional file 7: Table S6. Pairwise Nei’s genetic distances between the durum wheat genetic groups based on 10 SSR markers. [file 12863_2021_958_MOESM7_ESM.docx]

**Table S6.** Pairwise Nei’s genetic distances between the durum wheat subpopulations based on 10 SSR markers.

| **ADMIX** | **G1** | **G10** | **G11** | **G2** | **G3** | **G4** | **G5** | **G6** | **G7** | **G8** | **G9** |  |
| --- | --- | --- | --- | --- | --- | --- | --- | --- | --- | --- | --- | --- |
| 0.000 |  |  |  |  |  |  |  |  |  |  |  | **ADMIX** |
| 0.453 | 0.000 |  |  |  |  |  |  |  |  |  |  | **G1** |
| 0.673 | 1.365 | 0.000 |  |  |  |  |  |  |  |  |  | **G10** |
| 0.512 | 1.184 | 1.063 | 0.000 |  |  |  |  |  |  |  |  | **G11** |
| 0.565 | 1.189 | 1.437 | 0.708 | 0.000 |  |  |  |  |  |  |  | **G2** |
| 0.653 | 1.606 | 1.560 | 0.630 | 1.019 | 0.000 |  |  |  |  |  |  | **G3** |
| 0.502 | 0.871 | 0.985 | 0.716 | 0.960 | 0.630 | 0.000 |  |  |  |  |  | **G4** |
| 0.468 | 0.738 | 2.416 | 0.769 | 0.934 | 1.351 | 0.934 | 0.000 |  |  |  |  | **G5** |
| 0.261 | 0.852 | 0.922 | 0.624 | 0.983 | 0.726 | 0.662 | 0.717 | 0.000 |  |  |  | **G6** |
| 0.715 | 1.039 | 2.319 | 0.740 | 1.200 | 0.692 | 1.350 | 0.873 | 0.706 | 0.000 |  |  | **G7** |
| 0.692 | 0.421 | 2.265 | 1.179 | 0.981 | 1.875 | 1.666 | 1.100 | 1.435 | 1.080 | 0.000 |  | **G8** |
| 0.423 | 1.432 | 1.857 | 1.337 | 0.993 | 1.271 | 1.667 | 0.805 | 0.902 | 0.923 | 1.132 | 0.000 | **G9** |
