# Supplementary material for: Morphological characterization and genetic diversity analysis of Tunisian durum wheat (Triticum turgidum var. durum) accessions
Source: BMC Genom Data. 2021 Feb 3;22:3. doi: 10.1186/s12863-021-00958-3 (PMC7860204; doi:10.1186/s12863-021-00958-3)
Supplement: Supplementary file 8 — Additional file 8: Table S7. Shannon-Weaver index (H′) estimated for the genetic clusters C1 and C2 defined by MSN analysis. [file 12863_2021_958_MOESM8_ESM.docx]

**Table S7.** Shannon-Weaver index (*H’*) estimated for the genetic clusters C1 and C2 defined by MSN analysis.

|  | **C1** | **C2** | **Mean** |
| --- | --- | --- | --- |
| **SC** | 0.48 | 0.44 | 0.46 |
| **SS** | 0.80 | 0.63 | 0.72 |
| **SD** | 0.94 | 0.75 | 0.84 |
| **SL** | 0.73 | 0.65 | 0.69 |
| **AL** | 0.75 | 0.38 | 0.57 |
| **AC** | 0.60 | 0.52 | 0.56 |
| **NS** | 0.83 | 0.48 | 0.65 |
| **GlC** | 0.74 | 0.87 | 0.80 |
| **GC** | 0.90 | 0.39 | 0.64 |
| **GSp** | 0.82 | 0.63 | 0.73 |
| **GSz** | 0.95 | 0.95 | 0.95 |
| **GN** | 0.74 | 0.62 | 0.68 |
| **Mean *H’*** | **0.77** | **0.61** | **0.69** |
| **SC :** spike color**; SS :** spike shape**; SD :** spike density**; SL :** spike length**; AL :** awn length**; AC :** awn color**; NS :** number of spikelets/spike**; GlC :** glume color**; GC :** grain color**; GSp :** grain shape**; GSz :** grain size **;**  **GN :** number of grains/spikelet | | | |
