# Supplementary material for: Morphological characterization and genetic diversity analysis of Tunisian durum wheat (Triticum turgidum var. durum) accessions
Source: BMC Genom Data. 2021 Feb 3;22:3. doi: 10.1186/s12863-021-00958-3 (PMC7860204; doi:10.1186/s12863-021-00958-3)
Supplement: Supplementary file 9 — Additional file 9: Table S8. Diversity indexes of the genetic clusters C1 and C2 defined by MSN analysis. [file 12863_2021_958_MOESM9_ESM.docx]

**Table S8.** Diversity indexes of the genetic clusters C1 and C2 defined by MSN analysis.

| **Cluster** | **N** | **Na** | **Ne** | ***I*** | ***Ho*** | ***He*** | ***Nm*** | **F*st*** |
| --- | --- | --- | --- | --- | --- | --- | --- | --- |
| **C1** | 121 | 11 (4.000) | 5.246 (2.713) | 1.722 (0.581) | 0.004 (0.004) | 0.740  (0.135) | |  |
| **C2** | 140 | 6  (4.000) | 2.350 (1.197) | 0.911 (0.654) | 0.004 (0.004) | 0.425  (0.293) | |  |
| **Total** | 261 | 8.500 (2.723) | 3.798 (1.471) | 1.316 (0.427) | 0.004 (0.002) | 0.583 (0.160) | 1.889 (1.576) | 0.256 |

**N:** number of accessions; **Na :** Number of different Alleles ; **Ne :** Number of Effective Alleles ; ***I* :** Shannon's Information Index ; ***Ho* :** Observed Heterozygosity ; ***He* :** Expected Heterozygosity ; ***Nm* :** gene flow ; **F*st* :** genetic differenciation
