# Supplementary material for: Morphological characterization and genetic diversity analysis of Tunisian durum wheat (Triticum turgidum var. durum) accessions
Source: BMC Genom Data. 2021 Feb 3;22:3. doi: 10.1186/s12863-021-00958-3 (PMC7860204; doi:10.1186/s12863-021-00958-3)
Supplement: Supplementary file 10 — Additional file 10: Table S9. Frequencies of the different phenotypic classes calculated for each trait by regions and by climatic stages. [file 12863_2021_958_MOESM10_ESM.docx]

**Table S9.** Frequencies of the different phenotypic classes calculated for the 12 morphological traits by regions and by climatic stages*

|  |  | **SC** | **SS** | **SD** | **SL** | **AL** | **AC** | **NS** | **GlC** | **GC** | **GSp** | **GSz** | **GN** |
| --- | --- | --- | --- | --- | --- | --- | --- | --- | --- | --- | --- | --- | --- |
| **Entire collection** |  | 0.81 | 0.28 | 1.61 | 0.25 | 0.06 | 0.16 | 0.08 | 0.62 | 0.46 | 0.13 | 0.17 | 0.02 |
|  |  | 0.15 | 0.17 | 0.27 | 0.39 | 0.39 | 0.07 | 0.46 | 0.22 | 0.06 | 0.56 | 0.40 | 0.58 |
|  |  | 0.04 | 0.03 | 0.30 | 0.36 | 0.55 | 0.06 | 0.46 | 0.16 | 0.31 | 0.31 | 0.43 | 0.40 |
|  |  |  | 0.40 | 0.28 |  |  | 0.71 |  |  | 0.18 |  |  |  |
|  |  |  | 0.12 |  |  |  |  |  |  |  |  |  |  |
| **Regions** | **Gabes** | 0.08 | 0.03 | 0.24 | 0.11 | 0.84 | 0.08 | 0.45 | 0.84 | 0.11 | 0.08 | 0.82 | 0.05 |
|  |  | 0.92 | 0.05 | 0.63 | 0.05 | 0.08 | 0.05 | 0.55 | 0.08 | 0.08 | 0.82 | 0.18 | 0.79 |
|  |  | 0.00 | 0.79 | 0.13 | 0.84 | 0.08 | 0.13 | 0.00 | 0.08 | 0.16 | 0.11 | 0.00 | 0.16 |
|  |  |  | 0.13 | 0.00 |  |  | 0.74 |  |  | 0.66 |  |  |  |
|  |  |  | 0.00 |  |  |  |  |  |  |  |  |  |  |
|  | **Kairouan** | 0.81 | 0.19 | 0.01 | 0.34 | 0.01 | 0.01 | 0.32 | 0.44 | 0.54 | 0.34 | 0.32 | 0.44 |
|  |  | 0.01 | 0.19 | 0.09 | 0.24 | 0.26 | 0.01 | 0.22 | 0.34 | 0.01 | 0.43 | 0.13 | 0.56 |
|  |  | 0.18 | 0.01 | 0.51 | 0.43 | 0.72 | 0.01 | 0.46 | 0.22 | 0.22 | 0.24 | 0.54 | 0.00 |
|  |  |  | 0.41 | 0.38 |  |  | 0.96 |  |  | 0.22 |  |  |  |
|  |  |  | 0.19 |  |  |  |  |  |  |  |  |  |  |
|  | **Mahdia** | 1.00 | 0.48 | 0.48 | 0.37 | 0.30 | 0.41 | 0.52 | 0.78 | 0.52 | 0.52 | 0.78 | 0.59 |
|  |  | 0.00 | 0.48 | 0.44 | 0.37 | 0.70 | 0.15 | 0.48 | 0.22 | 0.26 | 0.48 | 0.22 | 0.41 |
|  |  | 0.00 | 0.04 | 0.07 | 0.26 | 0.00 | 0.44 | 0.00 | 0.00 | 0.22 | 0.00 | 0.00 | 0.00 |
|  |  |  | 0.00 | 0.00 |  |  | 0.00 |  |  | 0.00 |  |  |  |
|  |  |  | 0.00 |  |  |  |  |  |  |  |  |  |  |
|  | **Medenine** | 0.73 | 0.59 | 0.36 | 0.14 | 0.27 | 0.18 | 0.64 | 0.73 | 0.64 | 0.27 | 0.55 | 0.05 |
|  |  | 0.27 | 0.23 | 0.64 | 0.73 | 0.14 | 0.27 | 0.36 | 0.27 | 0.27 | 0.14 | 0.45 | 0.50 |
|  |  | 0.00 | 0.18 | 0.00 | 0.14 | 0.59 | 0.55 | 0.00 | 0.00 | 0.09 | 0.59 | 0.00 | 0.45 |
|  |  |  | 0.00 | 0.00 |  |  | 0.00 |  |  | 0.00 |  |  |  |
|  |  |  | 0.00 |  |  |  |  |  |  |  |  |  |  |
|  | **Sousse** | 1.00 | 1.00 | 0.00 | 1.00 | 0.00 | 1.00 | 0.00 | 1.00 | 0.00 | 0.00 | 0.00 | 0.00 |
|  |  | 0.00 | 0.00 | 0.00 | 0.00 | 1.00 | 0.00 | 0.00 | 0.00 | 0.00 | 0.00 | 0.00 | 0.00 |
|  |  | 0.00 | 0.00 | 0.00 | 0.00 | 0.00 | 0.00 | 1.00 | 0.00 | 1.00 | 1.00 | 1.00 | 1.00 |
|  |  |  | 0.00 | 1.00 |  |  | 0.00 |  |  | 0.00 |  |  |  |
|  |  |  | 0.00 |  |  |  |  |  |  |  |  |  |  |
| **Climatic stages** | **Low semi-arid** | 0.47 | 0.61 | 0.36 | 1.00 | 0.28 | 0.56 | 0.39 | 0.83 | 0.39 | 0.39 | 0.58 | 0.44 |
|  |  | 0.53 | 0.36 | 0.33 | 0.00 | 0.53 | 0.11 | 0.61 | 0.17 | 0.44 | 0.61 | 0.42 | 0.56 |
|  |  | 0.00 | 0.03 | 0.31 | 0.00 | 0.19 | 0.33 | 0.00 | 0.00 | 0.17 | 0.00 | 0.00 | 0.00 |
|  |  |  | 0.00 | 0.00 |  |  | 0.00 |  |  | 0.00 |  |  |  |
|  |  |  | 0.00 |  |  |  |  |  |  |  |  |  |  |
|  | **Mid-arid** | 0.15 | 0.25 | 0.53 | 0.85 | 0.08 | 0.10 | 0.58 | 0.80 | 0.30 | 0.15 | 0.72 | 0.05 |
|  |  | 0.58 | 0.10 | 0.38 | 0.15 | 0.33 | 0.15 | 0.42 | 0.15 | 0.15 | 0.57 | 0.28 | 0.68 |
|  |  | 0.27 | 0.57 | 0.08 | 0.00 | 0.58 | 0.08 | 0.00 | 0.05 | 0.10 | 0.28 | 0.00 | 0.27 |
|  |  |  | 0.08 | 0.00 |  |  | 0.67 |  |  | 0.45 |  |  |  |
|  |  |  | 0.00 |  |  |  |  |  |  |  |  |  |  |
|  | **High-arid** | 0.01 | 0.19 | 0.01 | 0.81 | 0.34 | 0.01 | 0.33 | 0.45 | 0.55 | 0.34 | 0.33 | 0.45 |
|  |  | 0.27 | 0.19 | 0.07 | 0.01 | 0.22 | 0.01 | 0.22 | 0.34 | 0.01 | 0.42 | 0.13 | 0.55 |
|  |  | 0.72 | 0.01 | 0.52 | 0.18 | 0.43 | 0.01 | 0.45 | 0.21 | 0.21 | 0.24 | 0.54 | 0.00 |
|  |  |  | 0.40 | 0.39 |  |  | 0.96 |  |  | 0.22 |  |  |  |
|  |  |  | 0.19 |  |  |  |  |  |  |  |  |  |  |

**SC :** spike color**; SS :** spike shape**; SD :** spike density**; SL :** spike length**; AL :** awn length**; AC :** awn color**; NS :** number of spikelets/spike**; GlC :** glume color**; GC :** grain color**; GSp :** grain shape**; GSz :** grain size **; GN :** number of grains/spikelet**;**

*** Phenotypic classes per trait have the same order as mentioned in Table S11.**
