# Supplementary material for: Morphological characterization and genetic diversity analysis of Tunisian durum wheat (Triticum turgidum var. durum) accessions
Source: BMC Genom Data. 2021 Feb 3;22:3. doi: 10.1186/s12863-021-00958-3 (PMC7860204; doi:10.1186/s12863-021-00958-3)
Supplement: Supplementary file 11 — Additional file 11: Table S10. Global Positioning System coordinates, number of durum wheat accessions and regions of the different collecting sites provided by The National Gene Bank. [file 12863_2021_958_MOESM11_ESM.docx]

**Table S10.** Global Positioning System coordinates, number of durum wheat accessions and regions of the different collecting sites provided by The National Gene Bank (BNG).

| **Region** | **Site** | **Longitude** | **Latitude** | **Number of accessions** | **Accessions NGB code** |
| --- | --- | --- | --- | --- | --- |
| **Kairouan** | Sbikha, Kondar | 10° 18′ 0.4″ E | 35° 55′ 57.62″ N | 9 | \| 14773, 14774, 14780, 14783, 14792, 14794, 14797, 14839, 14841 \| \| --- \| |
|  | Sbikha, Nadhour | 10° 5′ 33.57″ E | 36° 7′ 18.58″ N | 4 | 14842, 14843, 14844, 14845 |
|  | Sbikha, Sisseb | 10° 21′ 69.4″ E | 35° 97′ 19.4″ N | 23 | \| 16154, 16155, 16160, 16161, 16168, 16176, 16178, 16179, 16180, 16181, 16182, 16183, 16184, 14748, 14749, 14753, 14759, 14764, 14767, 14769, 14770, 14771, 14772 \| \| --- \| |
|  | El Guefaî, Sbikha | 9° 93′ 62.7″ E | 35° 97′ 23.8″ N | 13 | \| 16185, 16186, 16193, 16194, 16206, 16207, 16210, 16211, 16214, 16215, 16216, 16217, 16218 \| \| --- \| |
|  | Sbikha | 10° 1′ 12″ E | 35° 55′ 48″ N | 12 | \| 16219, 16220, 16231, 16244, 16245, 16255, 16256, 16267, 16268, 16278, 16288, 16297 \| \| --- \| |
|  | Sidi El Heni, direction Kairouan | 10° 18′ 34.93″ E | 35° 40′ 54.56″ N | 7 | \| 16105, 16106, 16113, 16114, 16145, 16146, 16150 \| \| --- \| |
| **Gabes** | Menzel Hbib | 9°36'22.3"E | 34°13'34.2"N | 31 | \| 14846, 14849, 14853, 14857, 14860, 14861, 14864, 14868, 14871, 14873, 14956, 14957, 14964, 15087, 15088, 15102, 15107, 15125, 15129, 15130, 15131, 15132, 15133, 15134, 15135, 15136, 15137, 15138, 15139, 15141, 15142 \| \| --- \| |
|  | Elmzaraa. El Hamma | 9°47'50.3"E | 33°53'13.7"N | 4 | \| 15168, 15169, 15171, 15173 \| \| --- \| |
|  | Aouledkhout. Ara ouali | 9° 91′ 30″ E | 33° 96′ 44″ N | 3 | \| 16369, 28566, 28569 \| \| --- \| |
| **Medenine** | Ejjoref. Djerba | 10°43'56.3"E | 33°41'38.8"N | 1 | 15441 |
|  | Elhnichrate. Zarzis | 11°04'14.0"E | 33°30'19.7"N | 16 | \| 15524, 15525, 15529, 15533, 15534, 15537, 15546, 15555, 15564, 15576, 15584, 15587, 15588, 15590, 15591, 15643 \| \| --- \| |
|  | Salb. à 10km de Zarzis | 11°01'03.7"E | 33°27'36.7"N | 5 | \| 15729, 15730, 15732, 15733, 15734 \| \| --- \| |
| **Mahdia** | Route Bou Merdes | 10° 43′ 48″ E | 35° 27′ 0″ N | 13 | \| 15902, 15903, 15915, 15928, 15929, 15942, 15953, 15954, 15965, 15966, 15971, 15972, 15973 \| \| --- \| |
|  | Lahkayma (Ouled Dhaouadi) | 10°45'39.1"E | 35°32'32.6"N | 3 | \| 15974, 16023, 16024 \| \| --- \| |
|  | Elmharza (Ouled el Haj Ennaas) | 10° 18′ 57.88″ E | 35° 29′ 28.75″ N | 10 | \| 16057, 16058, 16065, 16066, 16073, 16074, 16078, 16081, 16084, 16085 \| \| --- \| |
|  | Elmharza (Ouled Mansour) | 10° 18′ 97.78″ E | 35° 49′ 65.75″ N | 1 | 16086 |
| **Sousse** | Henchet el Magroun. Msaken | 10° 34′ 42.15″ E | 35° 43′ 49.76″ N | 9 | 16087, 16088, 16091, 16092,  16095, 16096, 16100, 16103,  16104 |
| **Unknown** | - | - | - | 140 | 28591, 28596, 28599, 28600, 28601, 28606, 28607, 28614, 28615, 28623, 28624, 28657, 28671, 28672, 28681, 28682,  28694, 28695, 28708, 28709,  28720, 28721, 28732, 28733, 28745, 28746, 28747, 28751,  28752, 28759, 28760, 28769, 28772, 28773, 28776, 28777,  28778, 28791, 28792, 28973, 28988, 28989, 28995, 28997, 29005, 29016, 29017, 29025, 29036, 29037, 29040, 29041,  29043, 29139, 29164, 29191,  29208, 29213, 29215, 29221,  29251, 29268, 29356, 29378,  29379, 29400, 29403, 29404,  29442, 29443, 29449, 29450,  29457, 29458, 29466, 29474,  29476, 29478, 29479, 29494,  29502, 29512, 29522, 29531,  29542, 29554, 29561, 29562,  29565, 29566, 29567, 29568,  29571, 29572, 29574, 29575,  29576, 29578, 29580, 29581,  29582, 29583, 29584, 29585,  29586, 29587, 29588, 29589,  29590, 29591, 29599, 29700,  29702, 29703, 29706, 29714,  29715, 29726, 29727, 29735,  29736, 29745, 29756, 49609,  49617, 49624, 49625, 49629, 49630, 49633, 49641, 49649,  49652, 49653, 49654, 49661,  49668, 49675, 49681, 49687 |
| **Total** | 17 | - | - | 304 |  |
