# Supplementary material for: Morphological characterization and genetic diversity analysis of Tunisian durum wheat (Triticum turgidum var. durum) accessions
Source: BMC Genom Data. 2021 Feb 3;22:3. doi: 10.1186/s12863-021-00958-3 (PMC7860204; doi:10.1186/s12863-021-00958-3)
Supplement: Supplementary file 12 — Additional file 12: Table S11. Descriptors used for estimating spike- and grain-based trait diversity in the durum wheat landraces. [file 12863_2021_958_MOESM12_ESM.docx]

**Table S11.** Descriptors used for estimating spike- and grain-based trait diversity in the durum wheat landraces.

| **Morphological trait** | **Observed phenotypic class** | **Note/code used** |
| --- | --- | --- |
| Spike color  **(SC)** | White  Light colored  Dark colored | 1  2  3 |
| Spike shape  **(SS)** | Pyramidal  With parallel edges  Cylindrical  Half cylindrical-Rectangular  Fusiform | 1  3  5  7  9 |
| Spike density  **(SD)** | Lax  Intermediate  Dense  Very dense | 3  5  7  9 |
| Spike length  **(SL)** | Short (< 6 cm)  Medium (6 - 9 cm)  Long (> 9 cm) | 1  3  5 |
| Awn length compared to the spike  **(AL)** | Shorter han the spike  Same length as the spike  Longer than the spike | 1  3  5 |
| Awn color  **(AC)** | White/White-yellow  Light tan  Tan  Black | 1  2  3  4 |
| Number of spikelets per spike  **(NS)** | Low (<15)  Intermediate (16 -22)  High (> 23) | 1  2  3 |
| Glume color  **(GlC)** | White  Tan-Red  Grey-Black | 1  2  3 |
| Grain color  **(GC)** | Yellow-white  Yellow-orange  Light brown  Brown | 1  2  3  5 |
| Grain shape  **(GSp)** | Slightly long  Moderately long  Elongate | 1  2  3 |
| Grain size  **(GSz)** | Small (< 0.3 cm)  Intermediate (0.3 - 0.5 cm)  Big (> 0.5 cm) | 3  5  7 |
| Number of grains per spikelet  **(GN)** | Low (< 2)  Medium (2-3)  High (> 3) | 1  2  3 |
